# Design, Synthesis and Antitumor Activity of Novel Selenium-Containing Tepotinib Derivatives as Dual Inhibitors of c-Met and TrxR

**DOI:** 10.3390/molecules28031304

**Published:** 2023-01-30

**Authors:** Jinhui Hu, Li Chen, Zhonghui Lu, Han Yao, Yunfei Hu, Luanqi Feng, Yanqing Pang, Jia-Qiang Wu, Zhiling Yu, Wen-Hua Chen

**Affiliations:** 1 School of Biotechnology and Health Sciences, Wuyi University, Jiangmen 529020, China; 2 School of Pharmaceutical Sciences, Sun Yat-sen University, Guangzhou 510006, China; 3 Department of Phase I Clinical Research Center, The Second Affiliated Hospital of Guangzhou University of Chinese Medicine, Guangzhou 510006, China; 4 Center for Cancer and Inflammation Research, School of Chinese Medicine, Hong Kong Baptist University, Kowloon Tong, Hong Kong, China

**Keywords:** dual target, anticancer agent, c-Met inhibitor, TrxR inhibitor

## Abstract

Cellular mesenchymal–epithelial transition factor (c-Met), an oncogenic transmembrane receptor tyrosine kinase (RTK), plays an essential role in cell proliferation during embryo development and liver regeneration. Thioredoxin reductase (TrxR) is overexpressed and constitutively active in most tumors closely related to cancer recurrence. Multi-target-directed ligands (MTDLs) strategy provides a logical approach to drug combinations and would adequately address the pathological complexity of cancer. In this work, we designed and synthesized a series of selenium-containing tepotinib derivatives by means of selenium-based bioisosteric modifications and evaluated their antiproliferative activity. Most of these selenium-containing hybrids exhibited potent dual inhibitory activity toward c-Met and TrxR. Among them, compound **8b** was the most active, with an IC_50_ value of 10 nM against MHCC97H cells. Studies on the mechanism of action revealed that compound **8b** triggered cell cycle arrest at the G_1_ phase and caused ROS accumulations by targeting TrxR, and these effects eventually led to cell apoptosis. These findings strongly suggest that compound **8b** serves as a dual inhibitor of c-Met and TrxR, warranting further exploitation for cancer therapy.

## 1. Introduction

Cellular mesenchymal–epithelial transition factor (c-Met), an oncogenic transmembrane receptor tyrosine kinase (RTK), plays an essential function in invasive growth during embryo development and liver regeneration [1,2]. c-Met signaling pathway has been implicated as a critical regulator for maintaining intracellular redox homeostasis and oxidative stress [3]. c-Met promotes the onset, proliferation, invasion and metastasis of hepatocellular carcinoma (HCC) [4]. It has been reported that tepotinib, a potent c-Met inhibitor, exhibits promising activity in advanced HCC with c-Met overexpression in clinical studies, indicating that c-Met may serve as a therapeutic target for HCC [5]. Recent studies have demonstrated that activation of c-Met can modulate the redox protective nuclear factor erythroid 2-related factor 2 (Nrf2)/heme oxygenase-1 (HO-1) and downregulate reactive oxygen species (ROS), eventually inhibiting the death of cancer cells [6]. Treatment combined with c-Met and HO-1 inhibitors can promote ROS-induced oxidative stress and markedly reduce tumor growth [7]. Hence, promoting intracellular oxidative stress might serve as a strategy for improving anticancer efficacy and overcoming the resistance to anticancer drugs.

Thioredoxin reductase (TrxR), a selenium (Se)-dependent enzyme, is one of the most important antioxidant systems in cellular physiology [8]. TrxR is markedly upregulated in HCC and plays a critical role in cancer progression, suggesting that TrxR may serve as a promising target for cancer therapy [9]. Burgeoning evidence indicates that TrxR inhibitors display potent anticancer efficacy by promoting apoptosis in tumor cells [10,11,12]. The effectiveness of the redox-modulating strategy by mean of TrxR inhibition has been validated in HCC tumor models [13,14]. Se plays a crucial role in numerous physiological processes within the body and is essential to human health [15]. Se supplementation alongside chemotherapeutic agents and nonsteroidal anti-inflammatory drugs (NSAIDs) was found to enhance the efficacy of anticancer drugs by modulating cellular redox status [16,17]. However, Se supplementation has a narrow therapeutic range and may be toxic at high doses [18].

**Figure 1:**
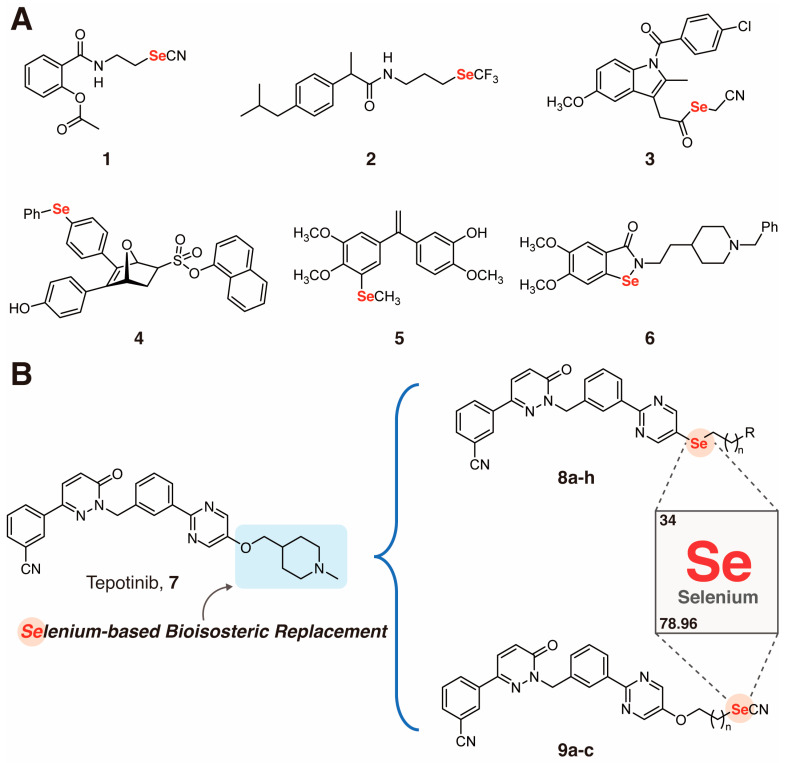
(**A**) Structures of Se-aspirin **1**, Se-flurbiprofen **2**, Se-indomethacin **3**, Se-OBHS (oxabicycloheptene sulfonate) **4**, Se-*iso*CA4 **5** and Se-donepezil **6**. (**B**) Design of multifunctional c-Met inhibitors **8a**–**h** and **9a**–**c** containing a Se moiety.

It is well recognized that Se bioisosteric modification of bioactive compounds is one practical strategy for designing multi-target-directed ligands (MTDLs) that could be therapeutically useful for treating cancer and other diseases [19]. Moreover, an MTDL-based strategy may adequately address the pathological complexity of cancer [20]. Several elegant examples of Se-containing compounds have been reported (Figure 1A). For example, a Se-aspirin analog (**1**) showed much higher antiproliferative activity against colorectal cancer (CRC) cells than fluorouracil [21]. The modification of flurbiprofen and ibuprofen frameworks with Se functionality (-SeCN and -SeCF_3_) was also explored [22,23]. Se-flurbiprofen and Se-ibuprofen (**2**) exhibited potent anti-inflammatory and anticancer activity. Se-indomethacin (**3**) was metabolized to release the parent indomethacin and Se fragment [24]. Recently, a multifunctional estrogen receptor (ER) modulator incorporating a phenylselenyl (PhSe) moiety into the skeleton of oxabicycloheptene sulfonate (OBHS), that is Se-OBHS (**4**), was reported to exhibit potent tumor suppression in tamoxifen-sensitive and -resistant tumor xenograft models [25]. Se-*iso*CA4 (**5**) showed potent antiproliferative activity, along with mitochondrial dysfunction [26]. Se-donepezil (**6**) exhibited potent inhibitory activity toward acetylcholinesterase (AChE) and mimicked glutathione peroxidase-like (GPx) activity [27].

Inspired by these findings, as well as by the potential advantages of MTDLs, we utilized a Se-based bioisostere strategy on tepotinib **7** and consequently designed a series of Se-containing compounds **8a**–**h** and **9a**–**c** (Figure 1B), with the aim of developing dual inhibitors of c-Met and TrxR. Such dual inhibitors may improve therapeutic effect while reducing toxicity to normal cells. Herein, we describe the synthesis of compounds **8a**–**h** and **9a**–**c** and systematic investigation into their antiproliferative activity, c-Met and TrxR inhibition, induction of ROS and cell cycle arrest/apoptosis-triggering ability.

## 2. Results and Discussion

### 2.1. Chemistry

**Scheme 1:**
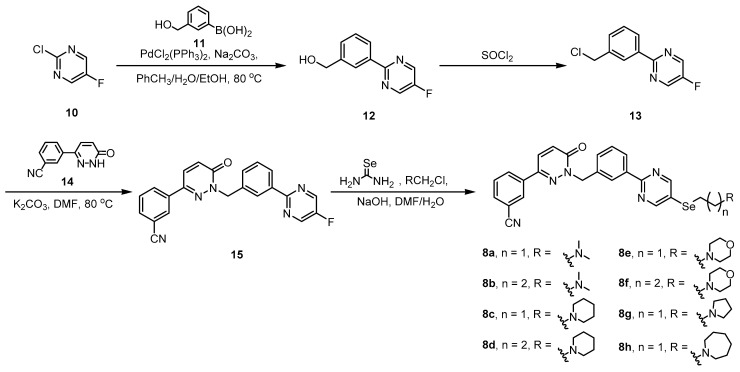
Synthesis of compounds **8a**–**h**.

The synthetic route of compounds **8a**–**h** is summarized in Scheme 1. Specifically, Suzuki–Miyaura cross-coupling of 2-chloro-5-fluoropyrimidine (**10**) with (3-(hydroxymethyl)phenyl)boronic acid (**11**) yielded compound **12**. Treatment of compound **12** with SOCl_2_ (to give chloride **13**) and subsequent substitution with 3-(6-oxo-1,6-dihydropyridazin-3-yl)benzonitrile (**14**) gave compound **15**. Finally, the reaction of selenourea with the corresponding chloroalkanes afforded the Se-containing intermediates, which were coupled with compound **15** in situ under basic conditions to afford compounds **8a**–**h**.

**Scheme 2:**
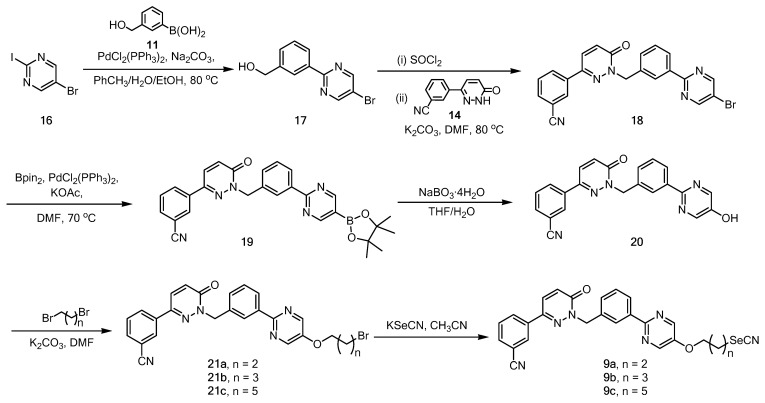
Synthesis of compounds **21a**–**c**.

The synthetic route of compounds **9a**–**c** is summarized in Scheme 2. Specifically, Suzuki–Miyaura cross-coupling of 5-bromo-2-iodopyrimidine (**16**) with compound **11** gave compound **17**. Treatment of compound **17** with SOCl_2_ and subsequent substitution with compound **14** gave compound **18**. The Miyaura borylation of compound **18** with bis(pinacolato)diboron yielded compound **19**, which was subject to sodium perborate oxidation to give compound **20**. The reaction of compound **20** with corresponding dibromoalkanes afforded compounds **21a**–**c**. Finally, the reaction of compounds **21a**–**c** with potassium selenocyanate provided compounds **9a**–**c**.

### 2.2. In Vitro Biological Evaluations

To assess the structure–activity relationship (SAR), compounds **8a**–**h** and **9a**–**c** were screened for their antiproliferative activity against human hepatocarcinoma cells (MHCC97H) using an MTT assay. Tepotinib **7** was used as a positive control. The antiproliferative activity of each compound is expressed as an IC_50_ value (Table 1). It can be seen that Se-bearing compounds generally exhibited potent antiproliferative activity, with the IC_50_ values ranging from 0.010 to 8.421 μM. Among them, 2-(dimethylamino)ethyl)selanyl-substituted compound **8b** showed the most potent antiproliferative activity. A series of hydrophilic side chains were introduced to the R position. As a consequence, the compounds having a side chain of two atoms, that is, compounds **8b**, **8d** and **8f**, had high activity. Compared with compound **8b**, replacement of the terminal dimethylamino substituent with *N*-heterocycle groups, including piperidine (**8c**,**d**), morpholine (**8e**,**f**), pyrrolidine (**8g**) and azepane (**8h**), led to a slight decrease in the antiproliferative activity. In addition, compounds **9a**–**c** bearing a selenocyanato linked to the aromatic skeleton through an alkoxy chain exhibited modest activity, and the activity was found to be dependent on the length of the alkoxy linkers, as supported by the successive decrease in the activity ongoing from **9a** to **9b** to **9c**.

### 2.3. c-Met and TrxR Inhibitory Effects in MHCC97H Cells

Based on the cytotoxicity results, we chose compounds **8a**, **8b**, **8d**, **8f** and **8g** to assess their inhibitory activity toward c-Met and TrxR by means of an ADP-Glo kinase assay kit and colorimetric assay (Table 1). Notably, compound **8b** displayed potent inhibition on both c-Met and TrxR, with the IC_50_ values being 0.010 and 0.099 μM, respectively. Compared with compound **8b**, replacement of the R substituent with piperidine (**8d**), morpholine (**8f**) and pyrrolidine (**8g**), respectively, led to a slight decrease in the inhibitory activity toward c-Met and TrxR. These results are in accordance with those obtained from the cytotoxicity assay.

### 2.4. Selectivity for Cancer Cells over Noncancer Cells

To evaluate the cytotoxic selectivity of compound 8b for cancer cells over noncancer cells, we determined the cytotoxicity of compound 8b against hepatocellular carcinoma cell line (HCCLM3) and human normal liver cells (LO2) using a standard MTT assay. The cytotoxicity and selectivity index (SI) of compound 8b are listed in Table 2. The 375-fold higher SI for LO2 than for cancer cells strongly suggests that compound 8b is more cytotoxic to hepatic cancer cells than to normal cells.

### 2.5. Effect of Compound **8b** on ROS Generation

**Figure 2:**
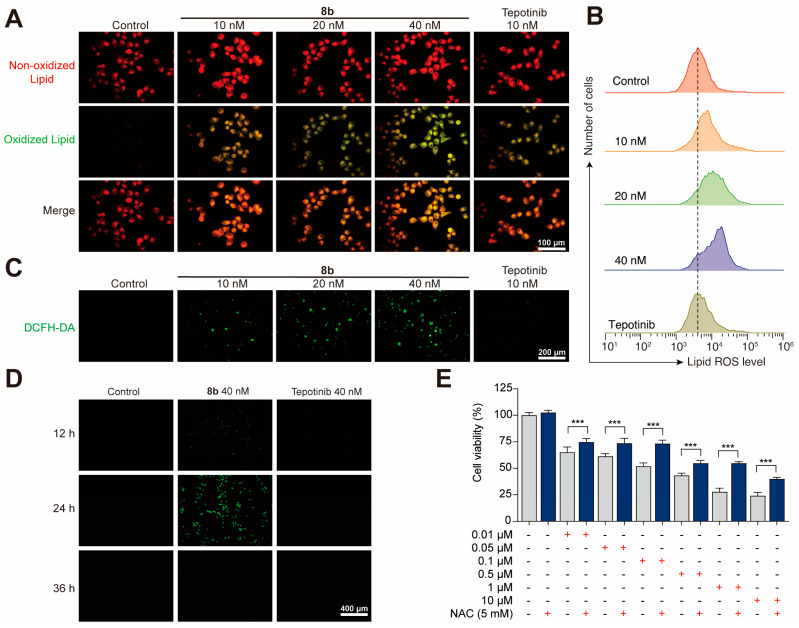
(**A**) Representative images for lipid peroxidation in MHCC97H cells using a C11-BODIPY-581/591 probe after incubation with compound **8b** (10, 20 and 40 nM) or tepotinib 7 (10 nM) for 24 h; (**B**) lipid ROS profiles were evaluated using the C11-BODIPY-581/591 probe with flow cytometry. (**C**,**D**) The production of intracellular ROS was measured with a DCF-DA probe and observed under an inverted fluorescence microscope. (**E**) The viability of MHCC97H cells induced by compound **8b** at the concentrations of 0.01, 0.05, 0.1, 0.5, 1 and 10 μM in the presence or absence of *N*-acetyl cysteine (NAC). *** *p* < 0.001.

It has been reported that TrxR inhibition is closely related to a significant increase in ROS production [10,28]. Thus, we detected the peroxidation of cellular lipid by using a fluorescent *C11-BODIPY* probe. Pronounced green fluorescence was observed in compound-8b-treated MHCC97H cells, indicating that this compound effectively causes the accumulation of cellular lipid peroxides (Figure 2A). The intracellular lipid ROS generation was further evaluated with flow cytometry. The significant lipid ROS formation appeared in the compound-8b-treated group in a dose-dependent manner compared with that of the tepotinib-7-treated group, which is attributed to the TrxR inhibitory activity of compound **8b** (Figure 2B). Moreover, the levels of intracellular ROS were measured using a 2,7-dichlorofluorescein diacetate (DCF-DA) probe. As a result, compound **8b** markedly induced the production of ROS, and the production of intracellular ROS was associated with TrxR inhibition (Figure 2C). To investigate the time-dependent effect of compound **8b** on the formation of ROS, we detected the levels of intracellular ROS by exposing compound **8b** to MHCC97H cells for 12, 24 and 36 h and analyzed the cells by using a DCF-DA probe. As shown in Figure 2D, the formation of ROS increased with time and reached its peak at 24 h. To assess whether the production of ROS plays a crucial role in the death of cancer cells induced by compound **8b**, we treated the MHCC97H cells in the presence or absence of a free radical scavenger (*N*-acetyl cysteine, NAC). As shown in Figure 2E, the cell death induced by compound **8b** in MHCC97H could be rescued by NAC. These results indicate that compound **8b** induced oxidative stress in MHCC97H cells by augmenting the levels of ROS and caused the death of cancer cells ultimately.

### 2.6. Cellular Apoptosis Analysis and Cell Cycle Study

**Figure 3:**
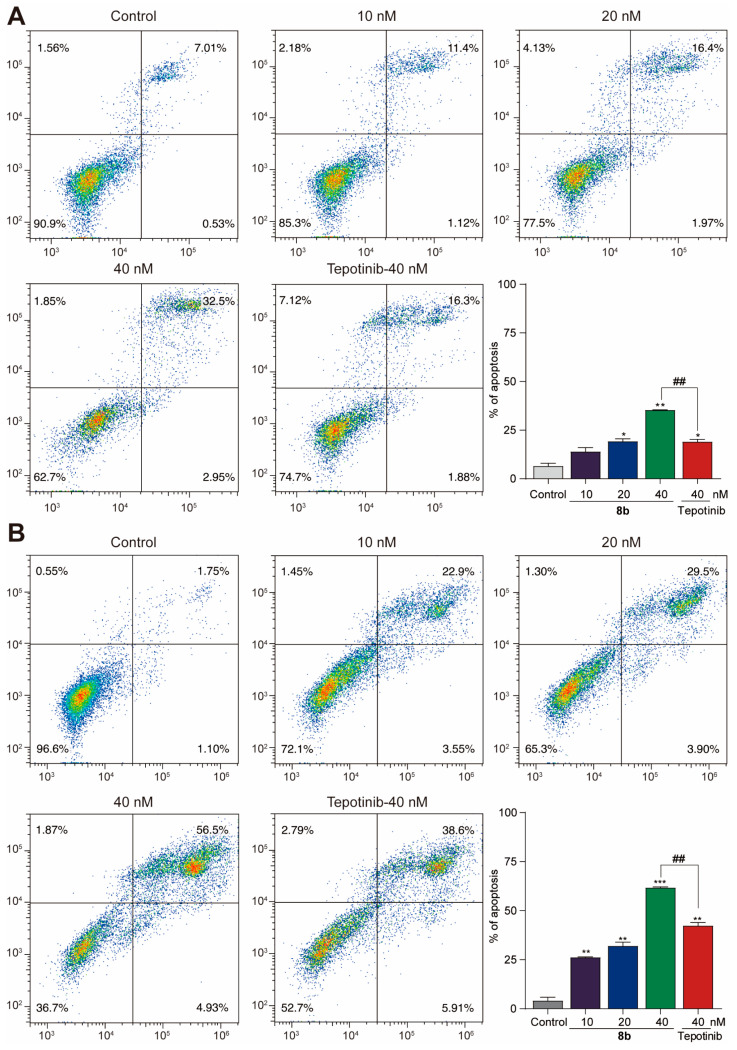
Effect of compound 8b on the apoptosis progression of MHCC97H cells. (**A**,**B**) Representative flow cytometric profiles showing that compound **8b** (10, 20 and 40 nM) induced the apoptosis of MHCC97H cells after 24 and 48 h, respectively. Histograms show the proportions of apoptotic cells. * *p* < 0.05, ** *p* < 0.01, *** *p* < 0.001 *vs*. control group; ## *p* < 0.01, compound **8b** (40 nM) vs. tepotinib **7** (40 nM).

**Figure 4:**
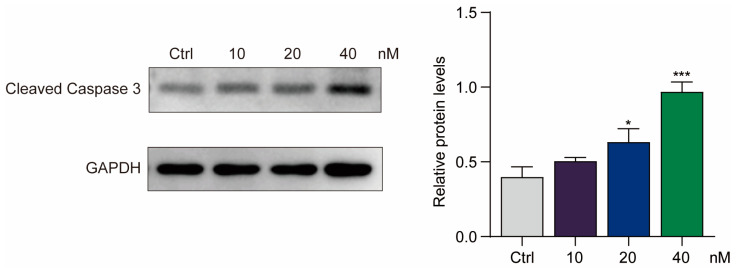
Effects of compound **8b** on the expression of cleaved caspase 3. MHCC97H cells were incubated with compound **8b** (10, 20 and 40 nM) for 24 h, followed by the Western blotting analysis of the expression of cleaved caspase 3. * *p* < 0.05, *** *p* < 0.001 vs. control.

It is reported that increasing the levels of intracellular ROS is highly related to the induction of cellular apoptosis [28]. As compound **8b** increased the production of ROS, we then investigated its effect on apoptosis in MHCC97H cells. Thus, MHCC97H cells were treated with this compound at varying concentrations for 24 h and 48 h, respectively, and analyzed by means of an Annexin V-FITC/PI assay. As shown in Figure 3, compared with the vehicle group, incubation with compound 8b led to a significant increase in the proportion of apoptotic cells. Specifically, upon the treatment with compound **8b** for 24 h, the proportions of apoptotic cells were 12.5% at 10 nM, 18.4% at 20 nM and 35.5% at 40 nM, respectively. After 48 h treatment with compound 8b, the corresponding proportions of apoptotic cells reached 26.5%, 33.4% and 61.4%, respectively. These results illustrated that compound **8b** effectively induced the apoptosis of MHCC97H cells in both dose- and time-dependent manners. Moreover, immunoblotting analysis demonstrated that compound **8b** markedly upregulated the expression of cleaved caspase 3, suggesting that ROS induced by compound 8b caused an increase in caspase-mediated apoptosis (Figure 4).

**Figure 5:**
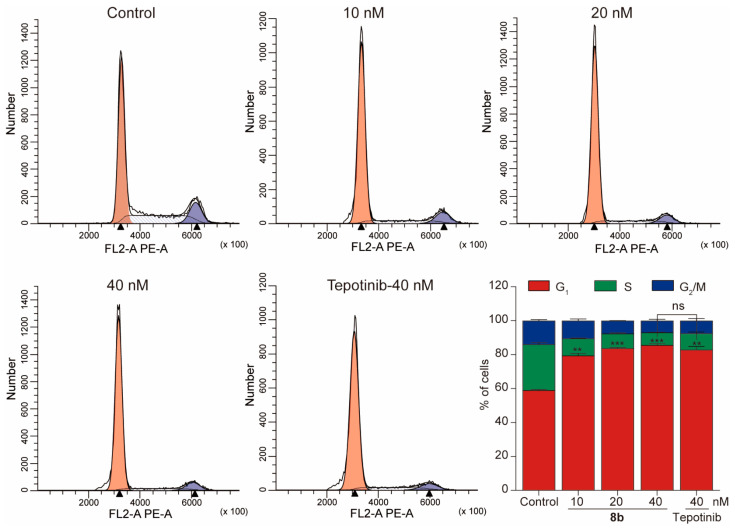
Compound **8b** induced the G_1_ phase arrest in MHCC97H cells. The MHCC97H cells were treated with compound **8b** (10, 20 and 40 nM) for 24 h, harvested, fixed with 70% ethanol, stained with PI and analyzed by flow cytometry. Histograms show the proportions of cell distribution. ** *p* < 0.01, *** *p* < 0.001.

It is known that inhibitors that block c-Met activation are able to arrest the cell cycle at the G_1_ phase [29]. Therefore, we performed a flow cytometric analysis to assess the cell-cycle distribution induced by compound **8b**, using propidium iodide (PI) staining in MHCC97H cells. As shown in Figure 5, the proportions of cells at the G_1_ phase in the presence of compound **8b** were 79.4% at 10 nM, 83.9% at 20 nM and 86.2% at 40 nM, respectively. This gradual accumulation of MHCC97H cells at the G_1_ phase with the increase in the concentration of compound **8b** strongly suggests that this compound induced cell cycle arrest at the G_1_ phase in MHCC97H cells.

In total, the above-mentioned findings clearly demonstrate several advantages of Se-containing compound **8b** for cancer treatment. Firstly, compound **8b** exhibited potent inhibitory activity toward TrxR, without any effect on its inhibitory activity toward c-Met. Secondly, compound **8b** markedly induced the formation of ROS and the production of intracellular ROS was associated with TrxR inhibition. In contrast, no obvious ROS formation was detected in the tepotinib-treated group. This result strongly suggests that the Se bioisosteric modification of tepotinib **7** was the cause of ROS generation induced by compound **8b**. Thirdly, the synergistic effect of TrxR and c-Met inhibition by compound **8b** might accelerate the redox imbalance in cancer cells. In combination with the dual inhibition of c-Met and TrxR, compound 8b exhibited more potent cell apoptosis than tepotinib. Fourthly, compound 8b is more cytotoxic to hepatic cancer cells than to normal cells. This selectivity is considered to be due to the Se replacement of tepotinib **7**, which is in agreement with previous reports that Se-containing compounds show high selectivity for cancer cells [30,31]. Hence, these advantages of compound **8b** ensure that Se replacement might be a promising strategy for the rational design of novel drugs in cancer treatment.

## 3. Materials and Methods

### 3.1. General Methods (Chemistry)

General methods are described in the Supplementary Material.

### 3.2. General Procedures for the Preparation of Compounds ***12*** and ***17***

A solution of compound 10 (or 16, 10 mmol), (3-(hydroxymethyl)phenyl)boronic acid (11, 12 mmol), PdCl_2_(PPh_3_)_2_ (5 mmol, 0.35 g) and Na_2_CO_3_ (20 mmol, 2.12 g) in a mixed solvent of PhCH_3_, H_2_O and EtOH (1/1/2, *v*/*v*/*v*, 20 mL) was stirred at 90 °C under nitrogen. After 18 h, the reaction mixture was cooled to room temperature and concentrated in vacuo. The resulting residue was partitioned between ethyl acetate and water. The organic layer was separated, washed with brine, dried over anhydrous Na_2_SO_4_ and concentrated. The residues were purified by silica gel chromatography using ethyl acetate/petroleum (8/1, *v*/*v*) as eluents to afford compound **12** (or **17**).

(3-(5-Fluoropyrimidin-2-yl)phenyl)methanol (**12**)

White solid, yield 55% (1.12 g) from compound 10 (10 mmol, 1.32 g). ^1^H NMR (500 MHz, CDCl_3_) δ 8.66 (s, 2H), 8.37 (s, 1H), 8.30 (dt, *J* = 6.7, 1.5 Hz, 1H), 7.54–7.46 (m, 2H), 4.79 (d, *J* = 6.0 Hz, 2H), 1.98 (t, *J* = 6.0 Hz, 1H) and ESI-MS *m/z*: 205.1 ([M+H]^+^).

(3-(5-Bromopyrimidin-2-yl)phenyl)methanol (**17**)

White solid, yield 64% (1.69 g) from compound 16 (10 mmol, 2.83 g). ^1^H NMR (500 MHz, CDCl_3_) δ 9.12 (s, 2H), 8.48–8.36 (m, 1H), 8.30–8.27 (m, 1H), 7.61–7.48 (m, 2H), 5.36 (t, *J* = 5.8 Hz, 1H), 4.65 (d, *J* = 5.8 Hz, 2H) and ESI-MS *m/z*: 265.0 ([M+H]^+^).

### 3.3. General Procedures for the Preparation of Compounds ***15*** and ***18***

A solution of compound 12 (or 17, 5 mmol) in SOCl_2_ (20 mL) was stirred at room temperature. After 2 h, the mixture was evaporated and the resulting residue was dissolved in anhydrous toluene. The solution was filtered and the filtrate was concentrated to afford chlorides, which were used for the next step without any further purification. A mixture of compound 14 (5.5 mmol, 1.08 g), K_2_CO_3_ (7.5 mmol, 1.04 g) and the above-mentioned chlorides in *N*,*N*-dimethylformamide (DMF, 10 mL) was stirred at 70 °C. After 18 h, the reaction mixture was cooled to room temperature and H_2_O was added. The mixture was extracted with ethyl acetate. The combined organic layers were washed with brine, dried over anhydrous Na_2_SO_4_ and evaporated. The residues were purified by silica gel chromatography using CH_2_Cl_2_/CH_3_OH (100/1, *v*/*v*) as eluents to afford compound **15** (or **18**).

3-(1-(3-(5-Fluoropyrimidin-2-yl)benzyl)-6-oxo-1,6-dihydropyridazin-3-yl)benzonitrile (**15**)

Yellow solid, yield 68% (1.30 g) from compound 12 (5 mmol, 1.02 g). ^1^H NMR (500 MHz, CDCl_3_) δ 8.68 (s, 2H), 8.60 (t, *J* = 1.5 Hz, 1H), 8.34 (dt, *J* = 7.8, 1.5 Hz, 1H), 8.16 (t, *J* = 1.5 Hz, 1H), 7.99 (dt, *J* = 7.8, 1.5 Hz, 1H), 7.70 (dt, *J* = 7.8, 1.5 Hz, 1H), 7.65 (d, *J* = 9.7 Hz, 1H), 7.62–7.55 (m, 2H), 7.48 (t, *J* = 7.8 Hz, 1H), 7.09 (d, *J* = 9.7 Hz, 1H), 5.51 (s, 2H) and ESI-MS *m/z*: 384.1 ([M+H]^+^).

3-(1-(3-(5-Bromopyrimidin-2-yl)benzyl)-6-oxo-1,6-dihydropyridazin-3-yl)benzonitrile (**18**)

Yellow solid, yield 57% (1.26 g) from compound 17 (5 mmol, 1.32 g). ^1^H NMR (500 MHz, DMSO-*d*_6_) δ 9.08 (s, 2H), 8.43 (d, *J* = 1.5 Hz, 1H), 8.40–8.35 (m, 1H), 8.30 (dt, *J* = 7.7, 1.5 Hz, 1H), 8.25 (dt, *J* = 8.0, 1.5 Hz, 1H), 8.15 (d, *J* = 9.7 Hz, 1H), 7.93 (dt, *J* = 7.8, 1.5 Hz, 1H), 7.73 (t, *J* = 7.8 Hz, 1H), 7.60 (dt, *J* = 7.8, 1.5 Hz, 1H), 7.50 (t, *J* = 7.8 Hz, 1H), 7.17 (d, *J* = 9.7 Hz, 1H), 5.46 (s, 2H) and ESI-MS *m/z*: 444.1 ([M+H]^+^).

### 3.4. General Procedures for the Preparation of Compounds ***8a***–***h***

A solution of selenourea (0.5 mmol, 61 mg) and corresponding chloroalkylamines (0.5 mmol) in anhydrous EtOH (2 mL) was stirred at 80 °C under nitrogen. After 7 h, the reaction mixture was cooled to room temperature and concentrated in vacuo. To a solution of the resulting residue in a mixed solvent of DMF and H_2_O (2/1, *v*/*v*, 3 mL) compound 15 (0.3 mmol, 115 mg) and NaOH (2.5 mmol, 111 mg) were added. The reaction mixture was stirred at 60 °C under nitrogen. After 3 h, the reaction mixture was extracted with ethyl acetate. The combined organic layers were washed with brine, dried over anhydrous Na_2_SO_4_ and evaporated. The residues were purified by silica gel chromatography using CH_2_Cl_2_/CH_3_OH (20/1, *v*/*v*) as eluents to afford compounds **8a**–**h**.

3-(1-(3-(5-((2-(Dimethylamino)ethyl)selanyl)pyrimidin-2-yl)benzyl)-6-oxo-1,6-dihydropyridazin-3-yl)benzonitrile (**8a**)

Pale yellow oil, yield 53% (82 mg) from compound 15 (0.3 mmol, 115 mg), selenourea (0.5 mmol, 61 mg) and 2-chloro-*N*,*N*-dimethylethan-1-amine (0.5 mmol, 54 mg). ^1^H NMR (400 MHz, CDCl_3_) δ 8.93 (s, 2H), 8.66 (t, *J* = 1.5 Hz, 1H), 8.38 (dt, *J* = 7.8, 1.5 Hz, 1H), 8.18 (t, *J* = 1.5 Hz, 1H), 7.98 (dt, *J* = 8.0, 1.5 Hz, 1H), 7.70 (dt, *J* = 7.8, 1.5 Hz, 1H), 7.66–7.60 (m, 2H), 7.58 (t, *J* = 8.0 Hz, 1H), 7.48 (t, *J* = 7.8 Hz, 1H), 7.07 (d, *J* = 9.7 Hz, 1H), 5.51 (s, 2H), 3.21–3.11 (m, 2H), 2.90–2.78 (m, 2H), 2.44 (s, 6H); ^13^C NMR (100 MHz, CDCl_3_) δ 163.0, 160.9, 159.4, 152.4, 142.2, 137.4, 136.3, 136.0, 132.5, 131.6, 130.8, 129.9, 129.8, 129.8, 129.4, 129.1, 129.0, 128.0, 118.4, 113.4, 58.8, 55.3, 44.3; HR-ESI for C_26_H_24_N_6_OSe ([M+H]^+^) Calcd: 517.1251; Found: 517.1235; Purity: 97.1% by HPLC, t_R_: 12.81 min.

3-(1-(3-(5-((3-(Dimethylamino)propyl)selanyl)pyrimidin-2-yl)benzyl)-6-oxo-1,6-dihydropyridazin-3-yl)benzonitrile (**8b**)

Pale yellow oil, yield 61% (97 mg) from compound 15 (0.3 mmol, 115 mg), selenourea (0.5 mmol, 61 mg) and 3-chloro-*N*,*N*-dimethylpropan-1-amine (0.5 mmol, 61 mg). ^1^H NMR (500 MHz, CDCl_3_) δ 8.89 (s, 2H), 8.60 (s, 1H), 8.36 (d, *J* = 7.7 Hz, 1H), 8.16 (s, 1H), 8.00 (d, *J* = 8.1 Hz, 1H), 7.71 (d, *J* = 7.7 Hz, 1H), 7.66 (d, *J* = 9.7 Hz, 1H), 7.63–7.55 (m, 2H), 7.49 (d, *J* = 7.7 Hz, 1H), 7.08 (d, *J* = 9.7 Hz, 1H), 5.51 (s, 2H), 2.99 (t, *J* = 7.0 Hz, 2H), 2.39 (t, *J* = 7.0 Hz, 2H), 2.21 (s, 6H), 1.89–1.86 (m, 2H); ^13^C NMR (125 MHz, CDCl_3_) δ 162.6, 160.5, 159.5, 142.3, 137.6, 136.2, 135.9, 132.6, 131.4, 130.8, 129.9, 129.8, 129.7, 129.5, 129.1, 128.8, 127.9, 124.3, 118.4, 113.4, 58.9, 55.6, 45.4, 28.0, 26.4; HR-ESI for C_27_H_26_N_6_OSe ([M+H]^+^) Calcd: 531.1408; Found: 531.1403; Purity: 97.3% by HPLC, t_R_: 18.92 min.

3-(6-Oxo-1-(3-(5-((2-(piperidin-1-yl)ethyl)selanyl)pyrimidin-2-yl)benzyl)-1,6-dihydropyridazin-3-yl)benzonitrile (**8c**)

Pale yellow oil, yield 62% from compound 15 (0.3 mmol, 115 mg), selenourea (0.5 mmol, 61 mg) and 1-(2-chloroethyl)piperidine (0.5 mmol, 74 mg). ^1^H NMR (400 MHz, CDCl_3_) δ 8.95 (s, 2H), 8.69 (t, *J* = 1.5 Hz, 1H), 8.40 (dt, *J* = 7.9, 1.5 Hz, 1H), 8.20 (t, *J* = 1.5 Hz, 1H), 7.99 (dt, *J* = 7.9, 1.5 Hz, 1H), 7.72 (dt, *J* = 7.7, 1.5 Hz, 1H), 7.66 (d, *J* = 9.7 Hz, 1H), 7.65–7.62 (m, 1H), 7.59 (d, *J* = 7.7 Hz, 1H), 7.49 (t, *J* = 7.7 Hz, 1H), 7.09 (d, *J* = 9.7 Hz, 1H), 5.52 (s, 2H), 3.36–3.26 (m, 2H), 3.04–2.94 (m, 2H), 2.89–2.56 (m, 4H), 1.92–1.78 (m, 4H), 1.60–1.49 (m, 2H); ^13^C NMR (125 MHz, CDCl_3_) δ 163.1, 160.9, 159.4, 142.2, 137.4, 136.3, 136.0, 132.5, 131.7, 130.8, 130.6, 129.9, 129.9, 129.8, 129.4, 129.2, 129.1, 128.0, 118.5, 113.4, 58.2, 55.3, 53.9, 53.7, 24.1, 23.1; HR-ESI for C_29_H_28_N_6_O_2_Se ([M+H]^+^) Calcd: 557.1564; Found: 557.1537; Purity: 96.2% by HPLC, t_R_: 18.67 min.

3-(6-Oxo-1-(3-(5-((3-(piperidin-1-yl)propyl)selanyl)pyrimidin-2-yl)benzyl)-1,6-dihydropyridazin-3-yl)benzonitrile (**8d**)

Pale yellow oil, yield 47% (80 mg) from compound 15 (0.3 mmol, 115 mg), selenourea (0.5 mmol, 61 mg) and 1-(3-chloropropyl)piperidine (0.5 mmol, 81 mg). ^1^H NMR (400 MHz, CDCl_3_) δ 8.88 (s, 2H), 8.61 (t, *J* = 1.5 Hz, 1H), 8.37 (dt, *J* = 7.8, 1.5 Hz, 1H), 8.14 (t, *J* = 1.5 Hz, 1H), 8.00 (dt, *J* = 8.0, 1.5 Hz, 1H), 7.69 (dt, *J* = 7.7, 1.5 Hz, 1H), 7.64 (d, *J* = 9.7 Hz, 1H), 7.62–7.59 (m, 1H), 7.58–7.55 (m, 1H), 7.47 (t, *J* = 7.7 Hz, 1H), 7.07 (d, *J* = 9.7 Hz, 1H), 5.51 (s, 2H), 2.98 (t, *J* = 7.0 Hz, 2H), 2.41–2.37 (m, 2H), 2.36–2.30 (m, 4H), 1.91 (q, *J* = 7.0 Hz, 2H), 1.56 (p, *J* = 5.6 Hz, 4H), 1.44–1.38 (m, 2H); ^13^C NMR (100 MHz, CDCl_3_) δ 162.5, 160.4, 159.4, 142.2, 137.7, 136.3, 136.0, 132.5, 131.3, 130.8, 129.9, 129.8, 129.7, 129.4, 129.1, 128.7, 127.9, 124.6, 118.4, 113.4, 58.5, 55.5, 54.7, 27.5, 26.8, 25.9, 24.4; HR-ESI for C_30_H_30_N_6_OSe ([M+H]^+^) Calcd: 571.1721; Found: 571.1708; Purity: 98.9% by HPLC, t_R_: 32.97 min.

3-(1-(3-(5-((2-Morpholinoethyl)selanyl)pyrimidin-2-yl)benzyl)-6-oxo-1,6-dihydropyridazin-3-yl)benzonitrile (**8e**)

Pale yellow oil, yield 66% (110 mg) from compound 15 (0.3 mmol, 115 mg), selenourea (0.5 mmol, 61 mg) and 4-(2-chloroethyl)morpholine (0.5 mmol, 75 mg). ^1^H NMR (400 MHz, CDCl_3_) δ 8.90 (s, 2H), 8.63 (s, 1H), 8.37 (d, *J* = 7.7 Hz, 1H), 8.16 (s, 1H), 7.99 (d, *J* = 7.9 Hz, 1H), 7.70 (d, *J* = 7.7 Hz, 1H), 7.64 (d, *J* = 9.7 Hz, 1H), 7.61 (d, *J* = 7.9 Hz, 1H), 7.57 (t, *J* = 7.9 Hz, 1H), 7.47 (t, *J* = 7.7 Hz, 1H), 7.07 (d, *J* = 9.7 Hz, 1H), 5.51 (s, 2H), 3.73–3.67 (m, 4H), 3.09 (t, *J* = 7.1 Hz, 2H), 2.74 (t, *J* = 7.1 Hz, 2H), 2.51–2.44 (m, 4H); ^13^C NMR (100 MHz, CDCl_3_) δ 162.6, 160.6, 159.4, 142.2, 137.6, 136.3, 136.0, 132.6, 131.4, 130.8, 129.9, 129.8, 129.7, 129.4, 129.1, 128.8, 127.9, 124.3, 118.4, 113.4, 66.8, 58.3, 55.4, 53.3, 25.9; HR-ESI for C_28_H_26_N_6_O_2_Se ([M+H]^+^) Calcd: 559.1357; Found: 559.1333; Purity: 95.6% by HPLC, t_R_: 7.47 min.

3-(1-(3-(5-((3-Morpholinopropyl)selanyl)pyrimidin-2-yl)benzyl)-6-oxo-1,6-dihydropyridazin-3-yl)benzonitrile (**8f**)

Colorless oil, yield 56% (96 mg) from compound 15 (0.3 mmol, 115 mg), selenourea (0.5 mmol, 61 mg) and 4-(3-chloropropyl)morpholine (0.5 mmol, 82 mg). ^1^H NMR (500 MHz, CDCl_3_) δ 8.91 (s, 2H), 8.65 (t, *J* = 1.5 Hz, 1H), 8.39 (dt, *J* = 7.8, 1.5 Hz, 1H), 8.18 (t, *J* = 1.5 Hz, 1H), 8.01 (dt, *J* = 8.0, 1.5 Hz, 1H), 7.72 (dt, *J* = 7.7, 1.5 Hz, 1H), 7.67 (d, *J* = 9.7 Hz, 1H), 7.63 (dt, *J* = 7.7, 1.5 Hz, 1H), 7.60 (t, *J* = 7.9 Hz, 1H), 7.50 (t, *J* = 7.7 Hz, 1H), 7.10 (d, *J* = 9.7 Hz, 1H), 5.53 (s, 2H), 3.74–3.67 (m, 4H), 3.03 (t, *J* = 7.0 Hz, 2H), 2.46 (t, *J* = 7.0 Hz, 2H), 2.44–2.38 (m, 4H), 1.92 (p, *J* = 7.0 Hz, 2H); ^13^C NMR (100 MHz, CDCl_3_) δ 162.5, 160.4, 159.4, 142.2, 137.6, 136.3, 136.0, 132.5, 131.4, 130.8, 129.9, 129.8, 129.7, 129.4, 129.1, 128.8, 127.9, 124.4, 118.4, 113.4, 66.9, 58.0, 55.5, 53.7, 27.1, 26.5; HR-ESI for C_29_H_28_N_6_O_2_Se ([M+H]^+^) Calcd: 573.1514; Found: 573.1500. Purity: 95.6% by HPLC, t_R_: 12.26 min.

3-(6-Oxo-1-(3-(5-((2-(pyrrolidin-1-yl)ethyl)selanyl)pyrimidin-2-yl)benzyl)-1,6-dihydropyridazin-3-yl)benzonitrile (**8g**)

Colorless oil, yield 66% (107 mg) from compound 15 (0.3 mmol, 115 mg), selenourea (0.5 mmol, 61 mg) and 1-(2-chloroethyl)pyrrolidine (0.5 mmol, 67 mg). ^1^H NMR (400 MHz, CDCl_3_) δ 8.93 (s, 2H), 8.66 (t, *J* = 1.5 Hz, 1H), 8.38 (dt, *J* = 7.8, 1.5 Hz, 1H), 8.18 (t, *J* = 1.5 Hz, 1H), 7.98 (dt, *J* = 8.1, 1.5 Hz, 1H), 7.70 (dt, *J* = 7.7, 1.5 Hz, 1H), 7.64 (d, *J* = 9.7 Hz, 1H), 7.62 (d, *J* = 7.7 Hz, 1H), 7.58 (t, *J* = 7.9 Hz, 1H), 7.48 (t, *J* = 7.7 Hz, 1H), 7.07 (d, *J* = 9.7 Hz, 1H), 5.51 (s, 2H), 3.24–3.18 (m, 2H), 3.06–3.00 (m, 2H), 2.89–2.74 (m, 4H), 1.96–1.89 (m, 4H); ^13^C NMR (100 MHz, CDCl_3_) δ 162.9, 160.8, 159.4, 142.2, 137.4, 136.3, 135.9, 132.5, 131.5, 130.8, 129.9, 129.8, 129.8, 129.5, 129.1, 128.9, 127.9, 123.2, 118.4, 113.3, 55.8, 55.3, 53.7, 25.0, 23.4; HR-ESI for C_28_H_26_N_6_OSe ([M+H]^+^) Calcd: 543.1408; Found, 543.1391; Purity: 95.4% by HPLC, t_R_: 11.77 min.

3-(1-(3-(5-((2-(Azepan-1-yl)ethyl)selanyl)pyrimidin-2-yl)benzyl)-6-oxo-1,6-dihydropyridazin-3-yl)benzonitrile (**8h**)

Pale yellow oil, yield 52% (89 mg) from compound 15 (0.3 mmol, 115 mg), selenourea (0.5 mmol, 61 mg) and 1-(2-chloroethyl)azepane (0.5 mmol, 81 mg). ^1^H NMR (400 MHz, CDCl_3_) δ 8.90 (s, 2H), 8.64 (t, *J* = 1.5 Hz, 1H), 8.38 (d, *J* = 8.0 Hz, 1 H), 8.17 (t, *J* = 1.5 Hz, 1H), 8.04–7.98 (m, 1H), 7.72 (d, *J* = 7.7 Hz, 1H), 7.66 (d, *J* = 9.7 Hz, 1H), 7.64–7.56 (m, 2H), 7.49 (t, *J* = 7.7 Hz, 1H), 7.09 (d, *J* = 9.7 Hz, 1H), 5.53 (s, 2H), 3.11 (t, *J* = 7.1 Hz, 2H), 2.89 (t, *J* = 7.1 Hz, 2H), 2.70–2.65 (m, 4H), 1.76–1.64 (m, 8H); ^13^C NMR (100 MHz, CDCl_3_) δ 162.4, 160.3, 159.4, 142.2, 137.7, 136.3, 136.0, 132.5, 131.3, 130.8, 129.9, 129.8, 129.7, 129.4, 129.1, 128.7, 127.8, 124.9, 118.4, 113.4, 57.6, 55.5, 55.2, 28.3, 27.7, 27.0; HR-ESI for C_30_H_30_N_6_OSe ([M+H]^+^) Calcd: 571.1721; Found: 571.1698; Purity: 95.4% by HPLC, t_R_: 19.94 min.

### 3.5. General Procedures for the Preparation of Compounds ***21a***–***c***

A mixture of compound 20 (1 mmol, 381 mg), corresponding dibromoalkane (1.5 mmol) and K_2_CO_3_ (1.5 mmol, 207 mg) in CH_3_CN (10 mL) was stirred at 85 ℃. After 10 h, the reaction mixture was cooled to room temperature and concentrated in vacuo. The resulting residue was partitioned between ethyl acetate and water. The organic layer was separated, washed with brine, dried over anhydrous Na_2_SO_4_ and concentrated. The residues were purified by silica gel chromatography using ethyl acetate/petroleum (4/1, *v*/*v*) as eluents to afford compounds **21a**–**c**.

3-(1-(3-(5-(3-Bromopropoxy)pyrimidin-2-yl)benzyl)-6-oxo-1,6-dihydropyridazin-3-yl)benzonitrile (**21a**)

Colorless oil, yield 79% (395 mg) from compound 20 (1 mmol, 381 mg) and 1,3-dibromopropane (1.5 mmol, 300 mg). ^1^H NMR (400 MHz, CDCl_3_) δ 8.59 (t, *J* = 1.8 Hz, 1H), 8.52 (s, 2H), 8.30 (dt, *J* = 8.0, 1.8 Hz, 1H), 8.18 (t, *J* = 1.8 Hz, 1H), 7.98 (dt, *J* = 8.0, 1.8 Hz, 1H), 7.70 (dt, *J* = 7.7, 1.8 Hz, 1H), 7.63 (d, *J* = 9.7 Hz, 1H), 7.60–7.53 (m, 2H), 7.45 (t, *J* = 7.7 Hz, 1H), 7.07 (d, *J* = 9.7 Hz, 1H), 5.51 (s, 2H), 4.28 (t, *J* = 6.0 Hz, 2H), 3.63 (t, *J* = 6.0 Hz, 2H), 2.38 (p, *J* = 6.0 Hz, 2H) and ESI-MS *m/z*: 503.4 ([M+H]^+^).

3-(1-(3-(5-(4-Bromobutoxy)pyrimidin-2-yl)benzyl)-6-oxo-1,6-dihydropyridazin-3-yl)benzonitrile (**21b**)

Colorless oil, yield 71% (366 mg) from compound 20 (1 mmol, 381 mg) and 1,4-dibromobutane (1.5 mmol, 321 mg). ^1^H NMR (400 MHz, CDCl_3_) δ 8.61 (s, 1H), 8.51 (s, 2H), 8.32 (d, *J* = 7.7 Hz, 1H), 8.20 (s, 1H), 8.00 (d, *J* = 7.6 Hz, 1H), 7.71 (d, *J* = 7.7 Hz, 1H), 7.65 (d, *J* = 9.7 Hz, 1H), 7.62–7.54 (m, 2H), 7.47 (t, *J* = 7.7 Hz, 1H), 7.09 (d, *J* = 9.7 Hz, 1H), 5.52 (s, 2H), 4.18 (t, *J* = 6.0 Hz, 2H), 3.53 (t, *J* = 6.0 Hz, 2H), 2.21–2.10 (m, 2H), 2.08–2.01 (m, 2H) and ESI-MS *m/z*: 516.1 ([M+H]^+^).

3-(1-(3-(5-((6-Bromohexyl)oxy)pyrimidin-2-yl)benzyl)-6-oxo-1,6-dihydropyridazin-3-yl)benzonitrile (**21c**)

Colorless oil, yield 75% (407 mg) from compound 20 (1 mmol, 381 mg) and 1,6-dibromohexane (1.5 mmol, 363 mg). ^1^H NMR (400 MHz, CDCl_3_) δ 8.58 (t, *J* = 1.7 Hz, 1H), 8.49 (s, 2H), 8.30 (dt, *J* = 7.7, 1.7 Hz, 1H), 8.17 (t, *J* = 1.7 Hz, 1H), 7.98 (dt, *J* = 7.9, 1.7 Hz, 1H), 7.69 (dt, *J* = 7.7, 1.7 Hz, 1H), 7.63 (d, *J* = 9.7 Hz, 1H), 7.60–7.52 (m, 2H), 7.45 (t, *J* = 7.7 Hz, 1H), 7.06 (d, *J* = 9.7 Hz, 1H), 5.50 (s, 2H), 4.12 (t, *J* = 6.3 Hz, 2H), 3.44 (t, *J* = 6.3 Hz, 2H), 1.98–1.89 (m, 2H), 1.89–1.78 (m, 2H), 1.58–1.49 (m, 4H) and ESI-MS *m/z*: 544.1 ([M+H]^+^).

### 3.6. General Procedures for the Preparation of Compounds ***9a***–***c***

A solution of **21a**–**c** (0.5 mmol) and KSeCN (0.75 mmol, 108 mg) in CH_3_CN (3 mL) was stirred at 85 °C. After 10 h, the reaction mixture was cooled to room temperature and concentrated in vacuo. The resulting residue was partitioned between ethyl acetate and water. The organic layer was separated, washed with brine, dried over anhydrous Na_2_SO_4_ and concentrated. The residues were purified by silica gel chromatography using ethyl acetate/petroleum (2/1, *v*/*v*) as eluents to afford compounds **9a**–**c**.

3-(6-Oxo-1-(3-(5-(3-selenocyanatopropoxy)pyrimidin-2-yl)benzyl)-1,6-dihydropyridazin-3-yl)benzonitrile (**9a**)

Pale yellow oil, yield 79% (209 mg); ^1^H NMR (400 MHz, CDCl_3_) δ 8.63 (t, *J* = 1.5 Hz, 1H), 8.54 (s, 2H), 8.33 (dt, *J* = 7.8, 1.5 Hz, 1H), 8.21 (t, *J* = 1.5 Hz, 1H), 7.99 (dt, *J* = 8.0, 1.5 Hz, 1H), 7.72 (dt, *J* = 7.7, 1.5 Hz, 1H), 7.65 (d, *J* = 9.7 Hz, 1H), 7.61–7.55 (m, 2H), 7.48 (t, *J* = 7.7 Hz, 1H), 7.09 (d, *J* = 9.7 Hz, 1H), 5.52 (s, 2H), 4.30 (t, *J* = 6.8 Hz, 2H), 3.32 (t, *J* = 6.8 Hz, 2H), 2.49 (5, *J* = 6.8 Hz, 2H); ^13^C NMR (100 MHz, CDCl_3_) δ 159.4, 157.8, 151.1, 144.0, 142.2, 137.8, 136.2, 136.0, 132.5, 130.8, 130.5, 129.9, 129.8, 129.8, 129.4, 129.0, 128.5, 127.4, 118.5, 113.4, 101.2, 66.8, 55.3, 30.2, 25.7; HR-ESI for C_26_H_20_N_6_O_2_Se ([M+H]^+^) Calcd: 529.0887; Found: 529.0877; Purity: 98.4% by HPLC, t_R_: 9.81 min.

3-(6-Oxo-1-(3-(5-(4-selenocyanatobutoxy)pyrimidin-2-yl)benzyl)-1,6-dihydropyridazin-3-yl)benzonitrile (**9b**)

Pale yellow oil, yield 81% (220 mg); ^1^H NMR (400 MHz, CDCl_3_) δ 8.60 (t, *J* = 1.5 Hz, 1H), 8.50 (s, 2H), 8.30 (dt, *J* = 7.8, 1.5 Hz, 1H), 8.18 (d, *J* = 1.5 Hz, 1H), 7.97 (dt, *J* = 7.8, 1.5 Hz, 1H), 7.69 (dt, *J* = 7.8, 1.5 Hz, 1H), 7.62 (d, *J* = 9.7 Hz, 1H), 7.59–7.52 (m, 2H), 7.45 (t, *J* = 7.8 Hz, 1H), 7.06 (d, *J* = 9.7 Hz, 1H), 5.50 (s, 2H), 4.17 (t, *J* = 5.9 Hz, 2H), 3.15 (t, *J* = 7.2 Hz, 2H), 2.22–2.12 (m, 2H), 2.07– 1.98 (m, 2H); ^13^C NMR (100 MHz, CDCl_3_) δ 159.4, 157.5, 151.4, 143.9, 142.2, 137.9, 136.1, 136.1, 132.5, 130.8, 130.4, 129.9, 129.8, 129.8, 129.4, 129.0, 128.5, 127.4, 118.5, 113.4, 101.2, 67.8, 55.3, 29.0, 28.6, 27.6; HR-ESI for C_27_H_22_N_6_O_2_Se ([M+H]^+^) Calcd: 543.1044; Found, 543.1041; Purity: 98.9% by HPLC, t_R_: 11.19 min.

3-(6-Oxo-1-(3-(5-((6-selenocyanatohexyl)oxy)pyrimidin-2-yl)benzyl)-1,6-dihydropyridazin-3-yl)benzonitrile (**9c**)

Pale yellow oil, yield 71% (202 mg); ^1^H NMR (400 MHz, DMSO-*d*_6_) δ 8.64 (s, 2H), 8.39–8.36 (m, 2H), 8.29–8.20 (m, 2H), 8.17 (d, *J* = 9.8 Hz, 1H), 7.93 (d, *J* = 7.7 Hz, 1H), 7.72 (t, *J* = 7.7 Hz, 1H), 7.50–7.44 (m, 2H), 7.16 (d, *J* = 9.8 Hz, 1H), 5.44 (s, 2H), 4.18 (t, *J* = 6.4 Hz, 2H), 3.10 (t, *J* = 7.3 Hz, 2H), 1.93–1.81 (m, 2H), 1.81–1.73 (m, 2H), 1.52–1.41 (m, 4H); ^13^C NMR (100 MHz, DMSO-*d*_6_) δ 159.2, 156.3, 152.0, 144.6, 142.4, 137.8, 137.4, 135.9, 133.3, 131.3, 130.8, 130.7, 130.6, 130.1, 129.9, 129.4, 127.4, 126.8, 119.0, 112.6, 105.0, 69.0, 55.1, 31.1, 30.2, 28.8, 28.5, 25.1; HR-ESI for C_29_H_26_N_6_O_2_Se ([M+H]^+^) Calcd: 571.1357; Found: 571.1366; Purity: 98.9% by HPLC, t_R_: 11.76 min.

## 4. Conclusions

In this study, a series of novel selenium-containing tepotinib derivatives were designed and synthesized as dual inhibitors of c-Met and TrxR. Among these compounds, compound **8b** exhibits potent antiproliferative activity against MHCC97H cells, with an IC_50_ value of 0.010 μM. In addition, compound **8b** induces the accumulation of intracellular ROS via inhibiting TrxR. Studies on the mechanism of action reveal that compound **8b** arrests the cell cycle at the G_1_ phase and induces cellular apoptosis. The present findings strongly suggest that compound **8b** may serve as a potent inhibitor of c-Met and TrxR and deserves further study.

## Figures and Tables

**Table 1:** In vitro antiproliferative activity of compounds **8a**–**h**, **9a**–**c** and tepotinib **7** against MHCC97H cell line, and inhibitory activity of some selected compounds and tepotinib **7** toward c-Met kinase and TrxR.

Compound	IC_50_ (μM) ^a^		
	MHCC97H ^b^	c-Met ^c^	TrxR ^d^
**8a**	0.027 ± 0.005	0.039 ± 0.008	0.421 ± 0.060
**8b**	0.010 ± 0.001	0.008 ± 0.002	0.099 ± 0.008
**8c**	0.075 ± 0.009	-	-
**8d**	0.038 ± 0.008	0.057 ± 0.006	0.654 ± 0.061
**8e**	0.042 ± 0.004	-	-
**8f**	0.029 ± 0.005	0.022 ± 0.003	0.117 ± 0.011
**8g**	0.063 ± 0.007	0.112 ± 0.023	0.800 ± 0.081
**8h**	0.374 ± 0.043	-	-
**9a**	1.215 ± 0.183	-	-
**9b**	5.748 ± 0.241	-	-
**9c**	8.421 ± 0.549	-	-
Tepotinib (**7**)	0.016 ± 0.001	0.009 ± 0.001	>10

^a^ IC_50_ values are indicated as the mean ± SD of three independent experiments. ^b^ The MHCC97H cells were treated with each compound for 48 h. Cell viability was measured by an MTT assay as described in the Supplementary Material. ^c^ C-Met kinase activity was measured by a c-Met kinase enzyme system with Kinase-Lumi kinase assay as described in the Supplementary Material. ^d^ The MHCC97H cells were treated with each compound for 24 h. The TrxR activity of the cell lysates was measured by a TrxR colorimetric assay as described in the Supplementary Material.

**Table 2:** In vitro antiproliferative activity of compound **8b** against MHCC97H, HCCLM3 and LO2 cell lines.

Compound	IC_50_ (μM) ^a^			SI ^b^
	MHCC97H	HCCLM3	LO2	
**8b**	0.010 ± 0.001	0.013 ± 0.005	4.314 ± 0.349	375
Tepotinib (**7**)	0.016 ± 0.001	0.020 ± 0.003	1.786 ± 0.208	99

^a^ IC_50_ values are indicated as the mean ± SD of three independent experiments. ^b^ SI is defined as a ratio of IC_50_ (noncancer)/average IC_50_ (hepatic cancer cell).

## Data Availability

All data are available from the authors.

## Supplementary Materials

The following supporting information can be downloaded at: https://www.mdpi.com/article/10.3390/molecules28031304/s1, NMR spectra of compounds **8a**–**h** and **9a**–**c**. HPLC chromatograms for compounds **8a**–**h** and **9a**–**c**.

## Sample Availability

Samples of compounds **8a**–**h** and **9a**–**c** are available from the authors.
