# Supplementary material for: Design, Synthesis and Antitumor Activity of Novel Selenium-Containing Tepotinib Derivatives as Dual Inhibitors of c-Met and TrxR
Source: Molecules. 2023 Jan 30;28(3):1304. doi: 10.3390/molecules28031304 (PMC9921947; doi:10.3390/molecules28031304)
Supplement: Supplementary file 1 [file molecules-28-01304-s001.zip › molecules-2144476-supplementary.pdf]

molecules

SUPPLEMENTARY MATERIALS

Design, Synthesis and Anti-Tumor Activity of Novel Selenium-Containing Tepotinib Derivatives as Dual Inhibitors of c-Met and TrxR

Jinhui Hu [1,*], Li Chen [1], Zhonghui Lu [1], Han Yao [2], Yunfei Hu [1], Luanqi Feng [1], Yanqing Pang [3], Jia-Qiang Wu [1], Zhiling Yu [4], Wen-Hua Chen [1,*]

[1] School of Biotechnology and Health Sciences, Wuyi University, Jiangmen 529020, China
[2] School of Pharmaceutical Sciences, Sun Yat-sen University, Guangzhou 510006, China
[3] Department of Phase I Clinical Research Center, The Second Affiliated Hospital of Guangzhou University of Chinese Medicine, Guangzhou, 510006, China
[4] Center for Cancer and Inflammation Research, School of Chinese Medicine, Hong Kong Baptist University, Kowloon Tong, Hong Kong, China

Table of Contents

1 Experimental Section 2
1.1 Chemistry 2
1.2 Biological Methods 2
1.2.1 Cell Culture and Cytotoxicity (MTT) Assay 2
1.2.2 c-Met Kinase Inhibitory Activity 2
1.2.3 Thioredoxin Reductase Activity Assay 3
1.2.4 Measurement of Lipid Peroxidation 3
1.2.5 Reactive Oxygen Species Assay 3
1.2.6 Cell Cycle Analysis 3
1.2.7 Cellular Apoptosis Analysis 4
1.2.8 Western Blotting Analysis 4
1.2.9 Statistical Analysis 4
2 NMR Spectra of Compounds **8a-h** and **9a-c** 5
3 HPLC Chromatograms of Compounds **8a-h** and **9a-c** 16
4 Uncropped and Unadjusted Images of Western Blot 22

1 Experimental Section

1.1 Chemistry

^{1}H NMR (500 or 400 MHz) and ^{13}C NMR (125 or 100 MHz) were recorded with a Bruker Avance III spectrometer. Low- and high-resolution mass spectra were obtained on an Agilent LC-MS 6120 instrument with an ESI and APCI mass selective detector and *Thermo Scientific Q Exactive-UltiMate3000* using electrospray ionization (ESI), respectively. The reactions were monitored by thin-layer chromatography (TLC) on silica gel plates and visualization was affected in an iodine chamber or at 254 nm. Flash column chromatography was performed using silica gel (200–300 mesh) purchased from Qingdao Haiyang Chemical Co. Ltd.

The purity (≥ 95%) of the samples was determined using an Agilent 1200 series HPLC system with a C18 column. Elution conditions: Mobile phase A, water containing 0.1 % Et_3N; Mobile phase B, acetonitrile; A:B = 70:30. The flow rate was 0.5 mL/min and the injection volume was 10 μL. The system operated at 25 °C. Peaks were detected at 254 nm. C18 column: Agilent Eclipse XDB-C18 5 μm, 4.6 mm × 150 mm column. The purity of compounds **8a-h** and **9a-c** is summarized in **Table S1**.

Table S1. The retention time and purity of compounds **8a-h** and **9a-c**

Compounds	Retention time (t_R)/min	% Purity
**8a**	12.81	97.1
**8b**	18.92	97.3
**8c**	18.67	96.2
**8d**	32.97	98.9
**8e**	7.47	95.6
**8f**	12.26	95.6
**8g**	11.77	95.4
**8h**	19.94	95.4
**9a**	9.81	98.4
**9b**	11.19	98.9
**9c**	11.76	98.9

1.2 Biological Methods

1.2.1 Cell Culture and Cytotoxicity (MTT) Assay

MHCC97H cells were purchased from FuHeng Cell Center, Shanghai, China. Cells were grown and cultured in DMEM medium (Procell, PM150210P) containing 10% (*v*/*v*) Fetal Bovine Serum (FBS) in humidified air environment containing 5% CO_2. The *in vitro* antiproliferative activity of each compound was assayed by means of an MTT method. In brief, cells were incubated at 37 °C for 24 h in 96-well plates at a density of 5×10^3/well. After removal of the media, culture media (100 μL) with 0.1% DMSO containing each compound at different concentrations was added to each well, and incubation continued at 37 °C for another 48 h. DMSO (0.1%) and tepotinib **7** were used as the negative and positive controls, respectively. MTT solution (5 mg/mL in PBS, Biofroxx, 0013B) was added and incubation continued for another 4 h. The optical density was detected with a microplate reader at 570 nm (BioTek, USA). The IC_{50} value of each compound was calculated using GraphPad Prism 7. All the experiments were repeated independently at least thrice.

1.2.2 c-Met Kinase Inhibitory Activity

Kinase reactions were conducted at 30 °C for 40 min. The reaction mixture (50 μL) consisted Tris (40 mM, pH 7.4), $MgCl_2$ (10 mM), BSA (0.1 mg/mL), DTT (1 mM), ATP (10 mM), Kinase and the enzyme-substrate (Promega, V3361). Each compound was diluted in 10% DMSO and the diluted solution (5 μL) was added to the reaction mixture (50 μL) so that the final concentration of DMSO was kept at 1% in all the reactions. The assay was performed using a Kinase-Lumi kinase assay kit (Beyotime, S0155S). The kinase activity was measured by quantitating the amount of ATP remaining in the solution.

1.2.3 Thioredoxin Reductase Activity Assay

A micro thioredoxin reductase (TrxR) colorimetric assay kit (Abbkine, CheKine™, KBT1650) was used to assess the efficacy of each tested compound (0.01, 0.1, 0.5, 1.0, and 5.0 μM) on thioredoxin reductase activity. MHCC97H cells were plated at 2×10^6/well, treated with each compound for 24 h, homogenized in assay buffer for 20 min on ice, and then centrifuged (10000× g) at 4 °C for 15 min. The protein contents of the supernatants were detected by a BCA protein assay (Beyotime, P0010S). d. The proteins samples (10 μg) were added to a buffer (100 μL) containing potassium phosphate (50 mM, pH 7.4), EDTA (1 mM) and NADPH (0.2 mM). Reaction was initiated by adding 5,5′-dithiobis(2-nitrobenzoic acid) (DTNB, 2 mM). The samples were analyzed at 412 nm every 60 s for 20 min using a Bio-Tek Synergy™ Microplate Reader.

1.2.4 Measurement of Lipid Peroxidation

A fluorescent probe C11-BODIPY (581/591) dye (ThermoFisher Scientific, #D3861) was used to detect the peroxidation of intracellular lipid according to the manufacturer's instructions.

(1) Flow cytometry analysis: MHCC97H cells were plated at 2×10^6/well, and treated with each compound for 24 h. The cells were then incubated with C11-BODIPY (5 μM) for 30 min at 37 °C in 24-well plates. All the cells were collected, spun down, washed with PBS, resuspended in phenol-red free medium, and finally analyzed using a CytoFLXE flow cytometer (Beckman Coulter, USA).

(2) Fluorescence imaging: MHCC97H cells were plated at 1×10^6/well, and treated with each compound for 24 h. The cells were incubated with C11-BODIPY (5 μM) for 30 min at 37 °C in 24-well plates, and then washed with PBS thrice. The excitation wavelength of C11-BODIPY581/591 was set at 488 nm (oxidized form) or 563 nm (nonoxidized form); oxidation of C11-BODIPY581/591 was revealed by the change of BODIPY fluorescence from red to green, and the fluorescence imaging was acquired by a microscope (Olympus, IX73P2F).

1.2.5 Reactive Oxygen Species Assay

The level of ROS was monitored by using a DCFH-DA assay (Beyotime, S0033M). MHCC97H cells were incubated at 37 °C for 24 h in 6 well-well plates at a density of 1×10^6/well. After removal of the media, culture media (1.5 mL) with 0.1% DMSO containing various concentrations of compound **8b** (10, 20 and 40 nM) was added to each well, and incubation continued at 37 °C for another 24 h. The cells were washed with PBS twice, and subjected to DCFH-DA (20 μM) for 20 min. Then, the cells were rinsed with PBS twice and fixed with a 4% PBS-buffered formalin solution. All the images were acquired with a fluorescence microscope (OLYMPUS, IX73P2F).

1.2.6 Cell Cycle Analysis

MHCC97H cells were incubated at 37 °C for 24 h in 6 well-well plates at a density of 2×10^6/well. After removal of the media, culture media (1.5 mL) with 0.1% DMSO containing various concentrations of compound **8b** (10, 20 and 40 nM) was added to each well, and incubation continued at 37 °C for another 24 h. The cells were collected by centrifugation, washed with PBS and fixed in ice-cold 70% ethanol at -20 °C for 2 h. After the removal of ethanol, the cells were resuspended in a staining buffer containing RNase and

propidium iodide (MA0334, Meilunbio, China) for 30 min. The DNA content of the cells was measured using a CytoFLXE flow cytometer (Beckman Coulter, USA).

1.2.7 Cellular Apoptosis Analysis

MHCC97H cells were incubated at 37 °C for 24 h in 6 well-well plates at a density of $2{\times}10^6$/well. After removal of the media, culture media (1.5 mL) with 0.1% DMSO containing various concentrations of compound **8b** (10, 20 and 40 nM) was added to each well, and incubation continued at 37 °C for another 24 h. The cells were harvested and washed with PBS twice. The washed cells were resuspended in a binding buffer and stained with Annexin V-FITC and PI (KGA105-KGA108, KeyGEN, China). Then, the cells were incubated at room temperature for 15 min without light exposure and then analyzed using a CytoFLXE flow cytometer (Beckman Coulter, USA).

1.2.8 Western Blotting Analysis

MHCC97H cells were cultured in a 6-well plate for 24 h, then the media containing compound **8b** of varying concentrations (10, 20 and 40 nM) were added to the petri dishes. The cells were then homogenized with lysis buffer. The protein concentrations were detected using a BCA Protein Assay Kit (Beyotime, P0010S), and their extracts were reconstituted within loading buffer (Beyotime, P0015A) and inactivated for 5 min at 100 °C. Subsequently, proteins (20 μg) were fractionated by 10% SDS-PAGE, and transferred to PVDF membranes. Then, their levels were determined with the appropriate dilution of primary antibodies, including GAPDH (Beyotime, AF1186) and cleaved caspase-3 (Beyotime, AF1150). The above primary antibodies were then incubated using HRP-conjugated secondary antibody, and immunoreactive bands were visible by a chemiluminescence reagent (Beyotime, A0208) and were estimated through densitometry with a Biomolecular Imager (Azure Biosystems, Sapphire RGBNIR). Intensities of the blots were quantified with ImageJ.

1.2.9 Statistical Analysis

All the statistical analyses were conducted using a GraphPad Prism software. Data are presented as mean ± SD from at least three independent experiments. The statistical significance of Differences in measured variables between two groups was assessed using an unpaired Student's *t* test and between multiple groups and conditions using one-way and two-way ANOVAs. A value of $p < 0.05$ was considered statistically significant (*$p < 0.05$; **$p < 0.01$; ***$p < 0.001$).

2 NMR Spectra of Compounds 8a-h and 9a-c

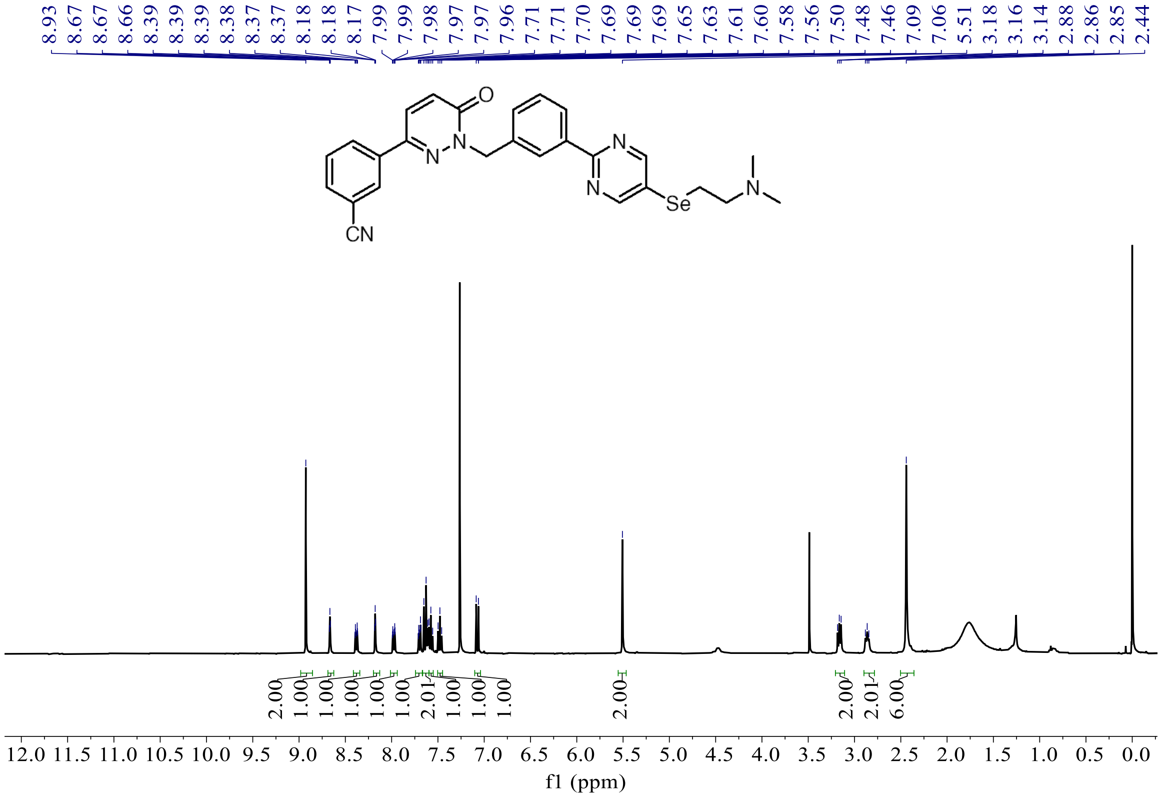

Figure S1. ^{1}H NMR of compound **8a** ($CDCl_3$, 400 MHz)

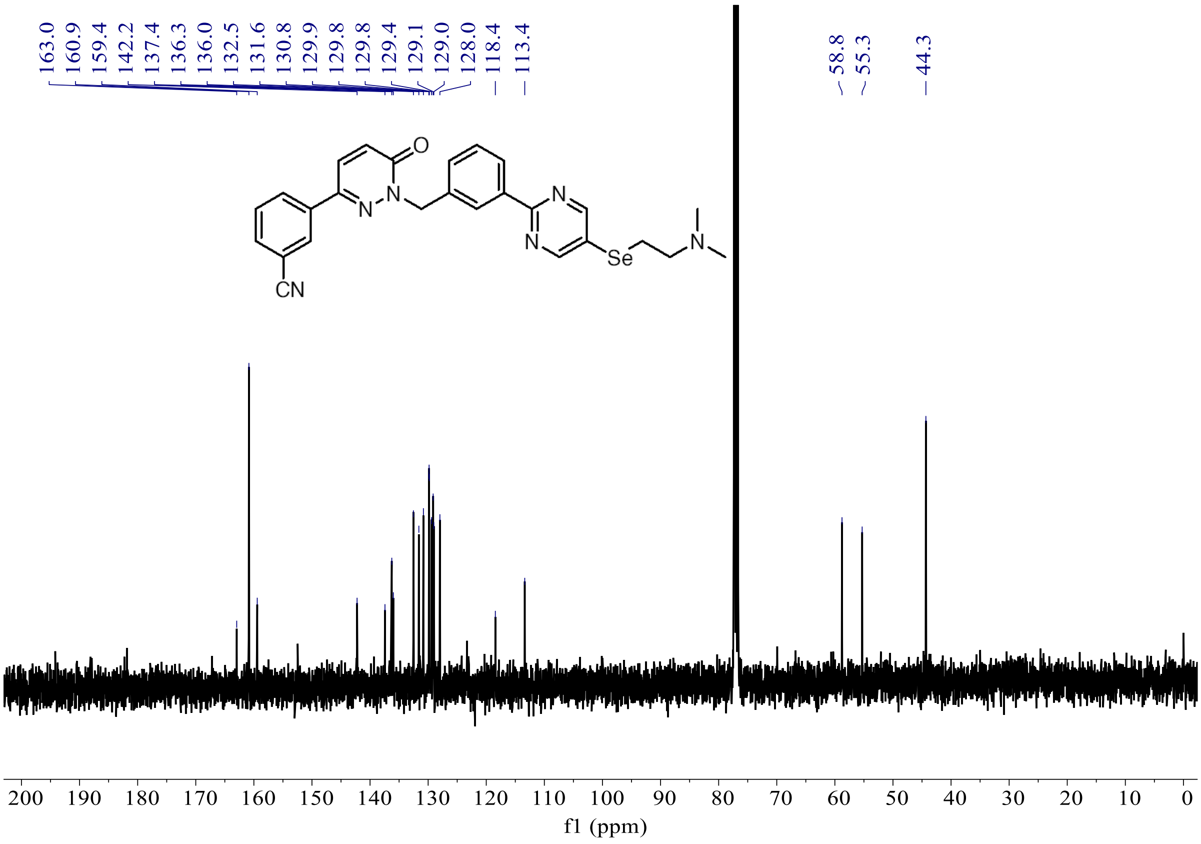

Figure S2. ^{13}C NMR of compound **8a** ($CDCl_3$, 100 MHz)

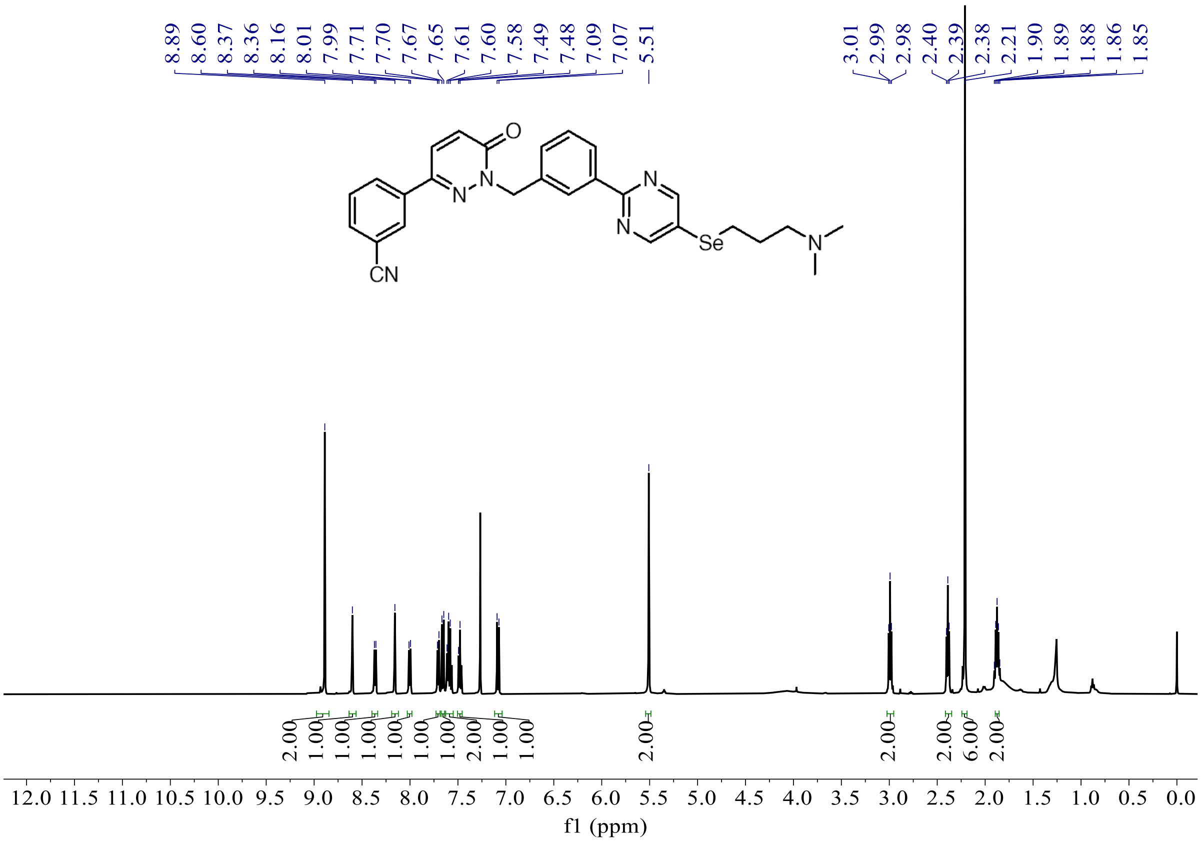

Figure S3. ^{1}H NMR of compound **8b** ($CDCl_3$, 500 MHz)

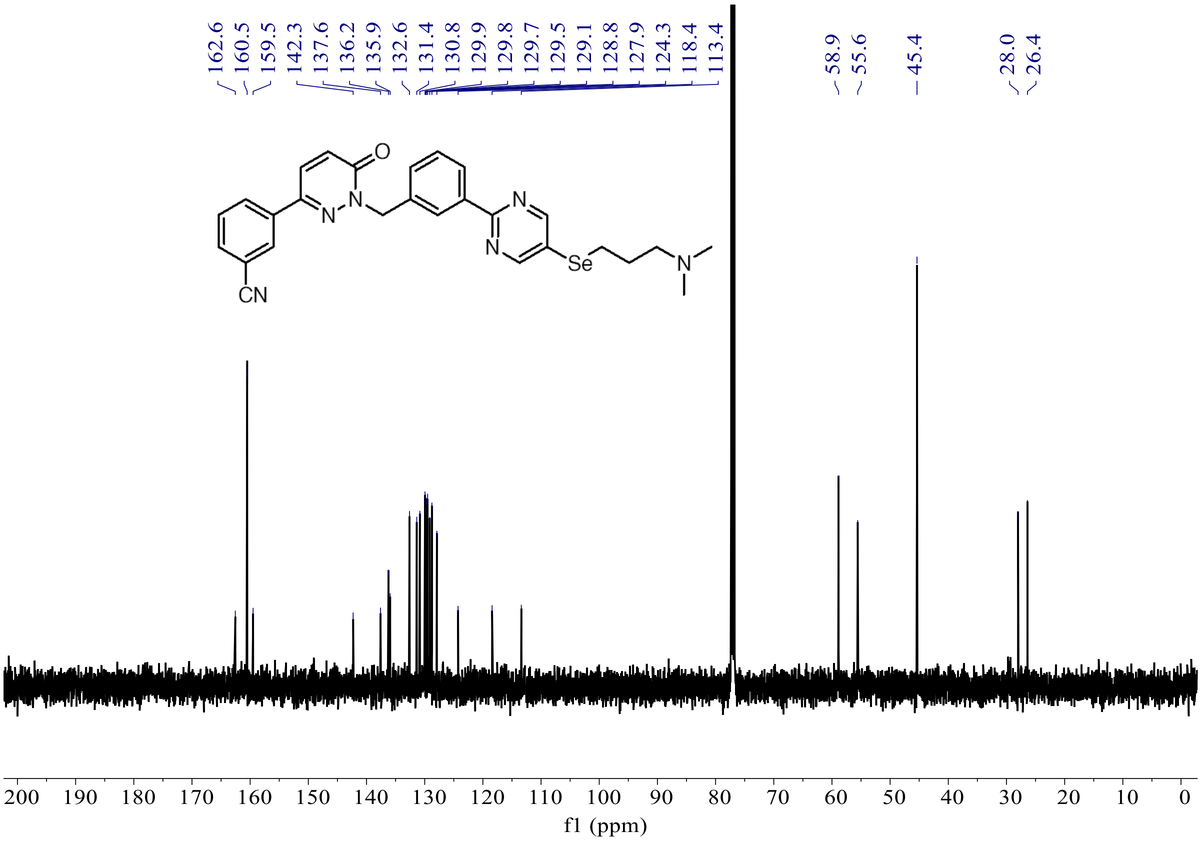

Figure S4. ^{13}C NMR of compound **8b** ($CDCl_3$, 125 MHz)

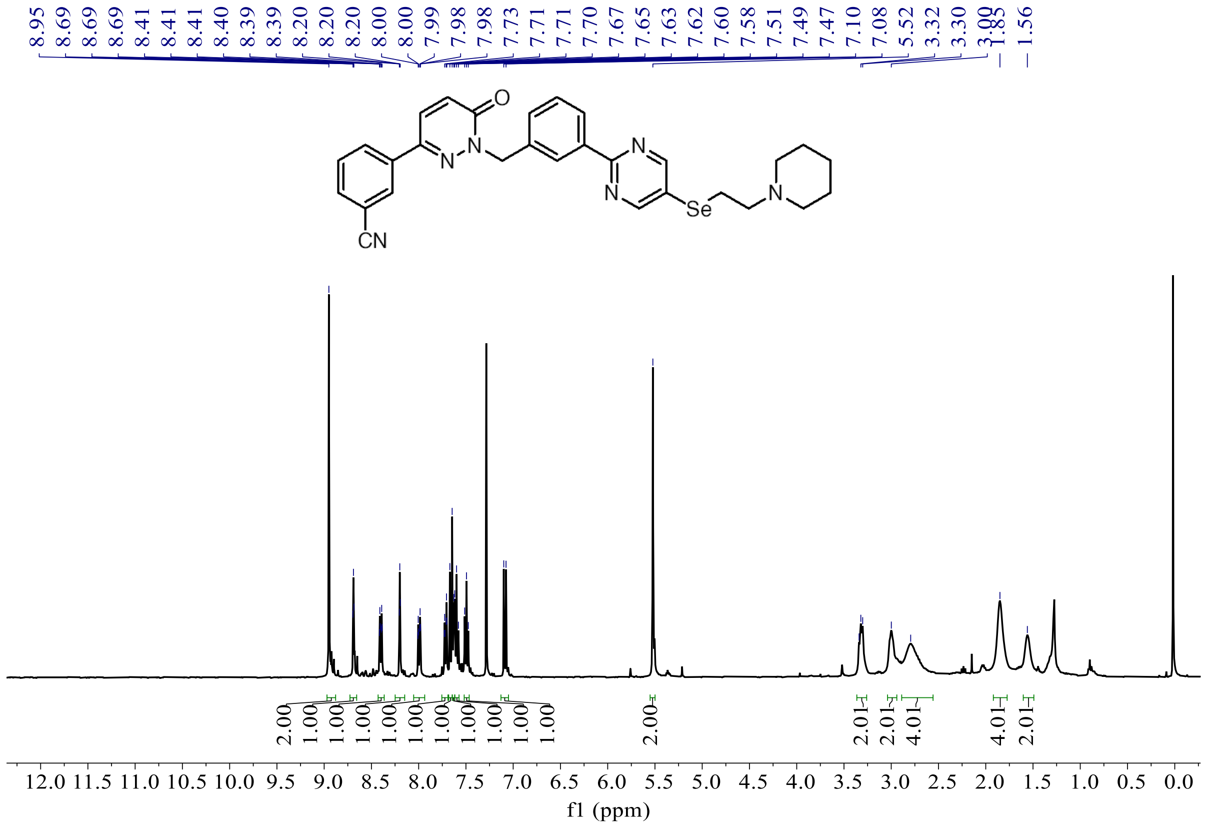

Figure S5. ^{1}H NMR of compound **8c** ($CDCl_3$, 400 MHz)

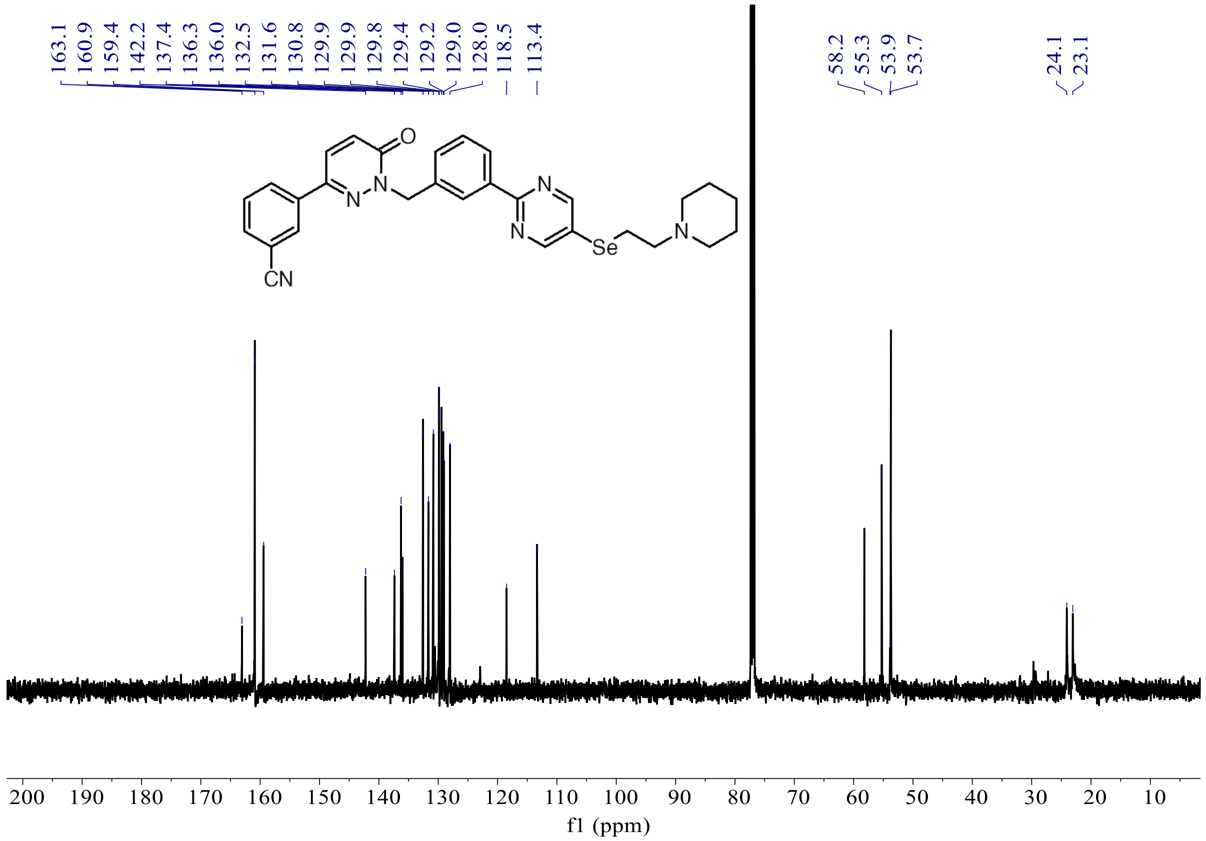

Figure S6. ^{13}C NMR of compound **8c** ($CDCl_3$, 125 MHz)

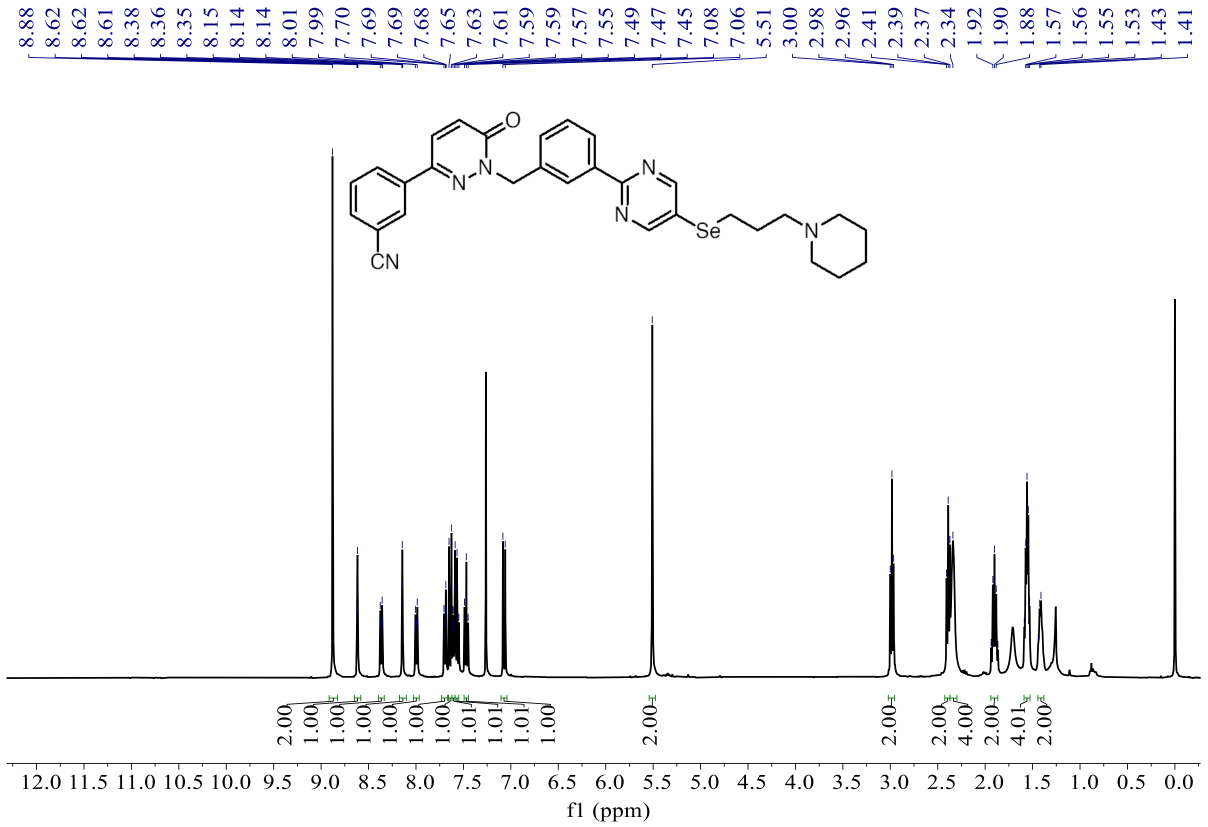

Figure S7. ^{1}H NMR of compound **8d** ($CDCl_3$, 400 MHz)

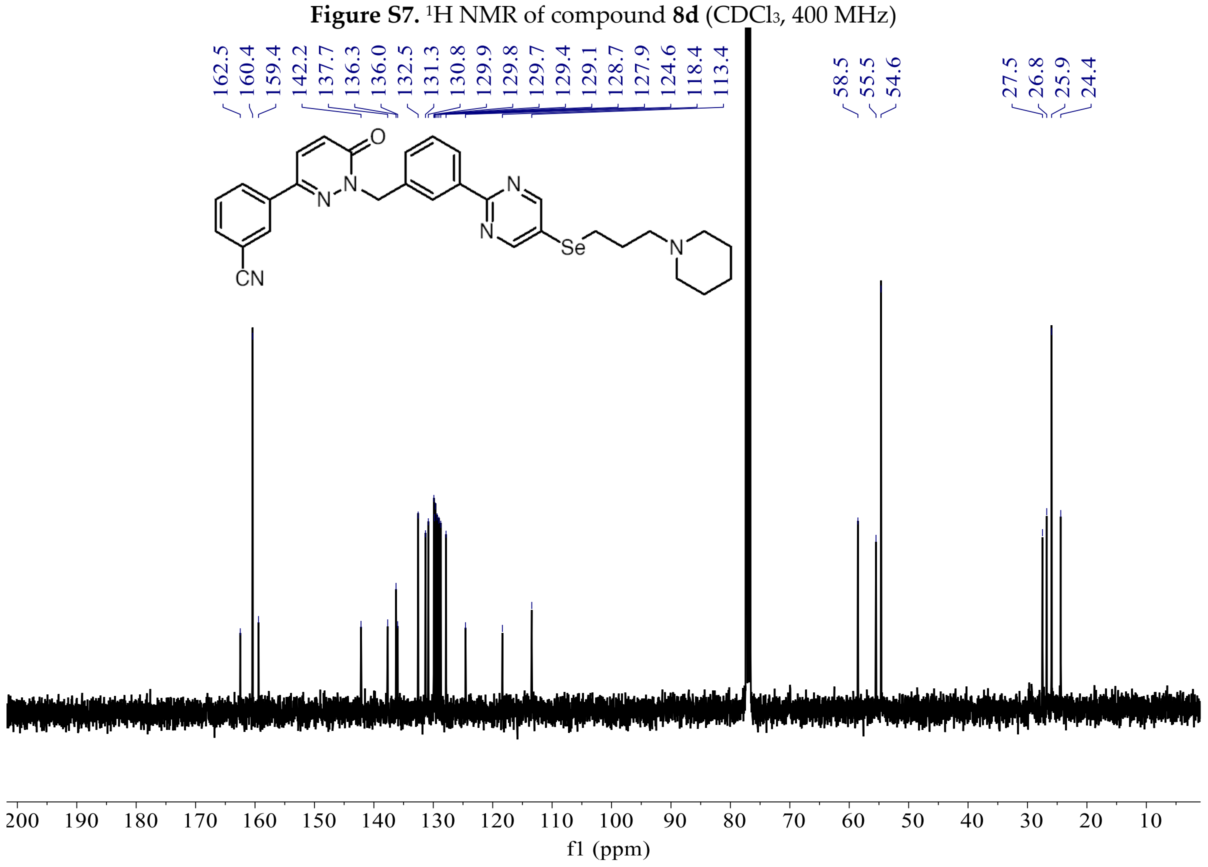

Figure S8. ^{13}C NMR of compound **8d** ($CDCl_3$, 100 MHz)

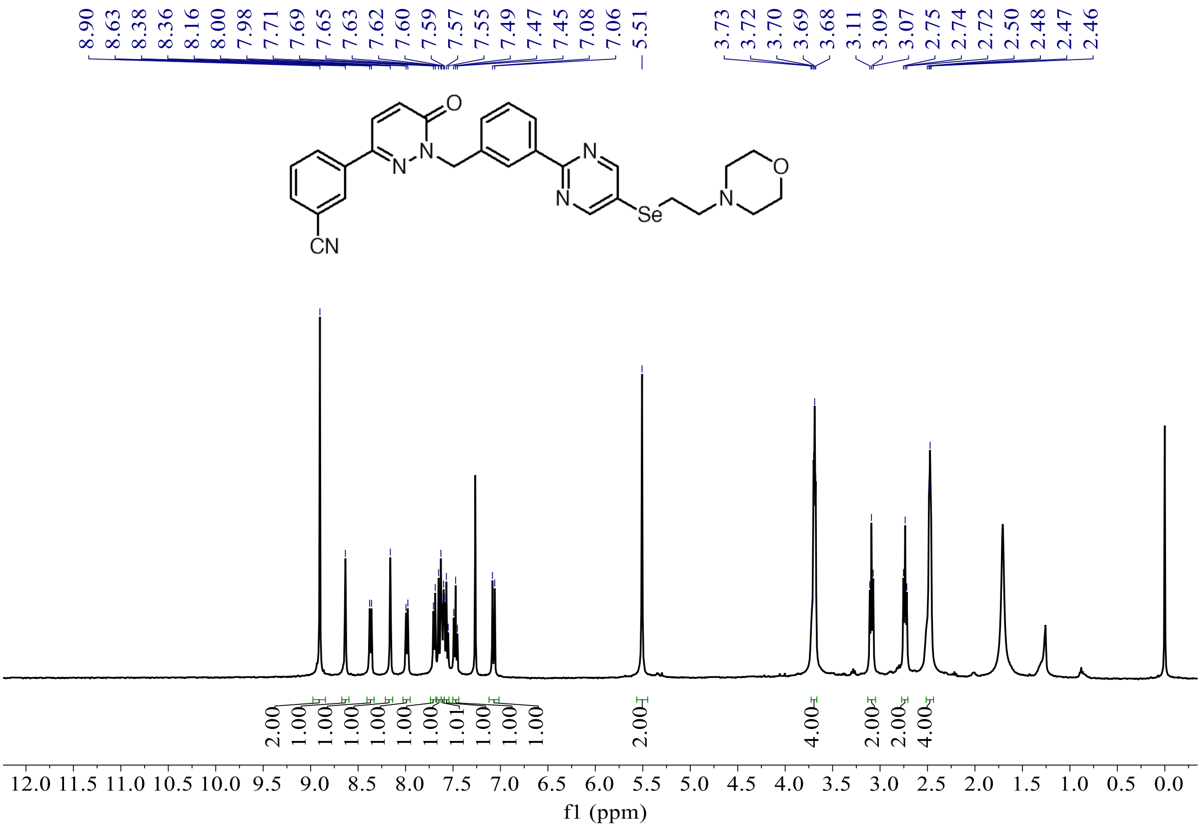

Figure S9. ^{1}H NMR of compound **8e** ($CDCl_3$, 400 MHz)

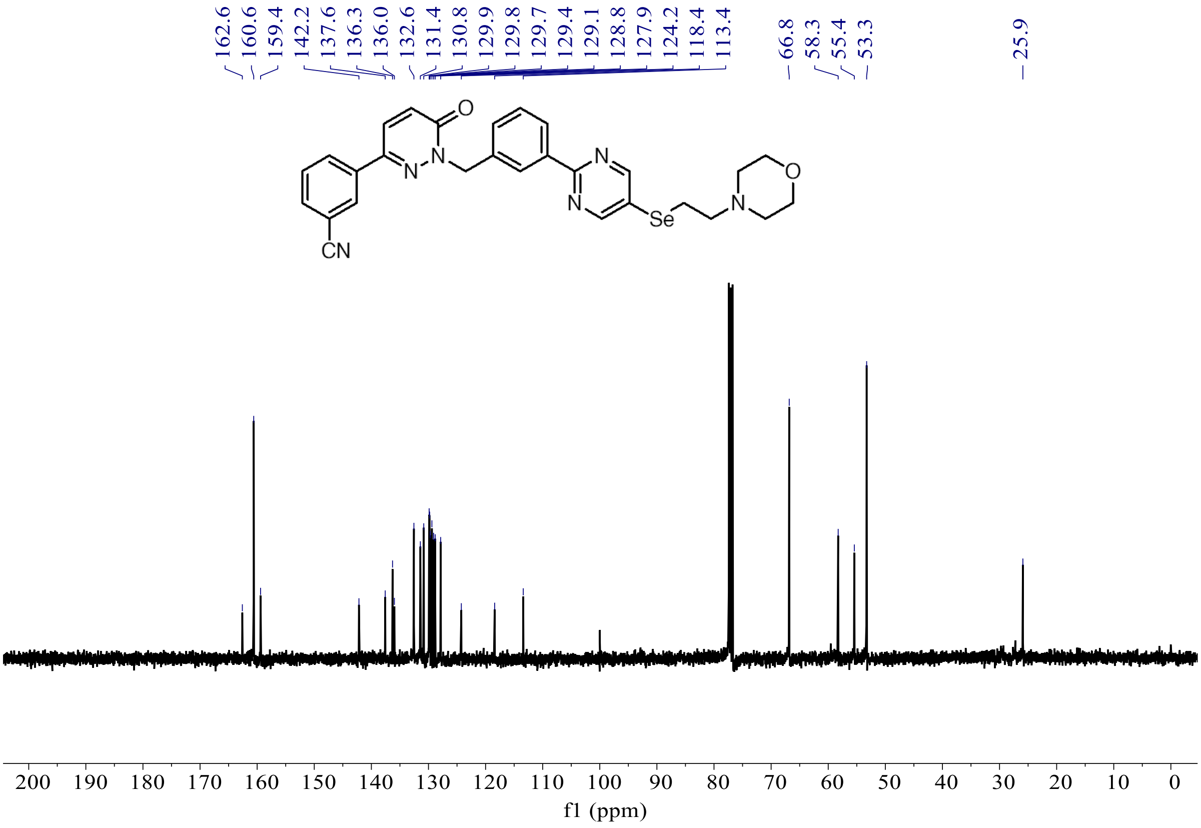

Figure S10. ^{13}C NMR of compound **8e** ($CDCl_3$, 100 MHz)

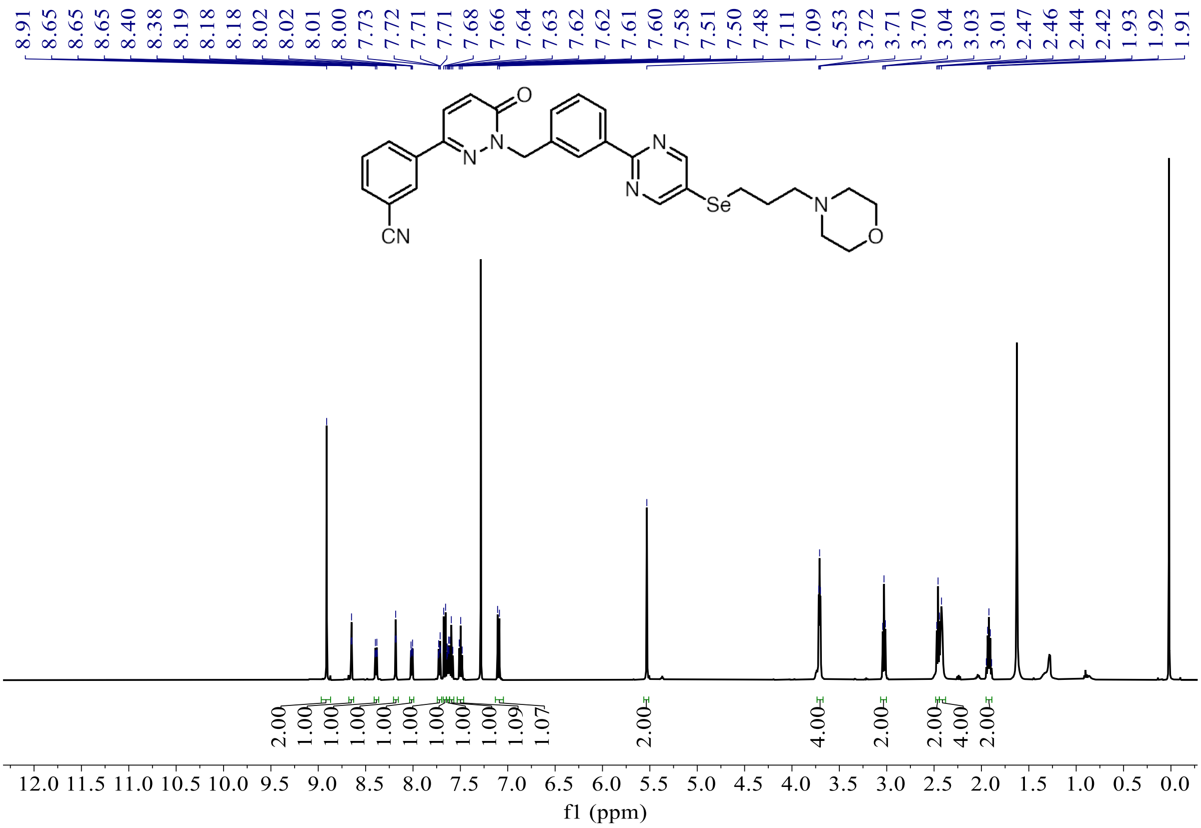

Figure S11. ^{1}H NMR of compound **8f** ($CDCl_3$, 500 MHz)

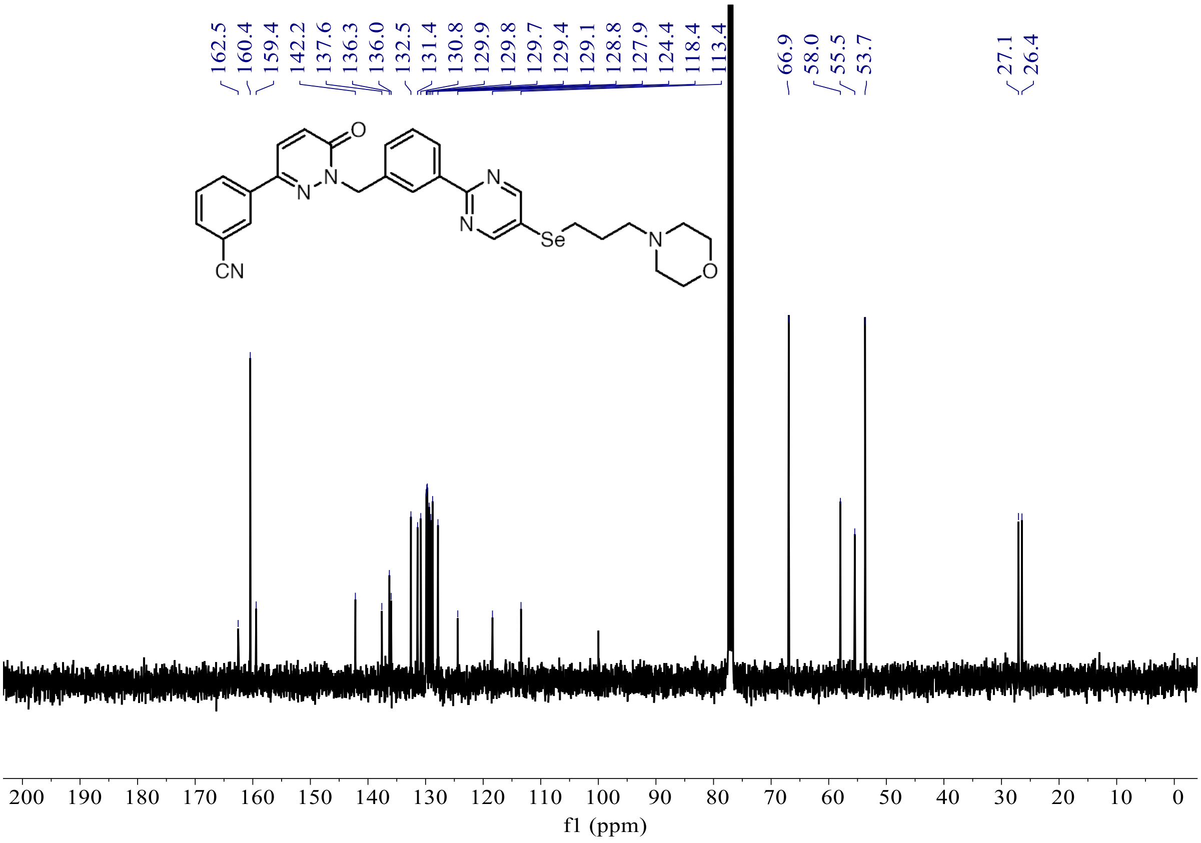

Figure S12. ^{13}C NMR of compound **8f** ($CDCl_3$, 100 MHz)

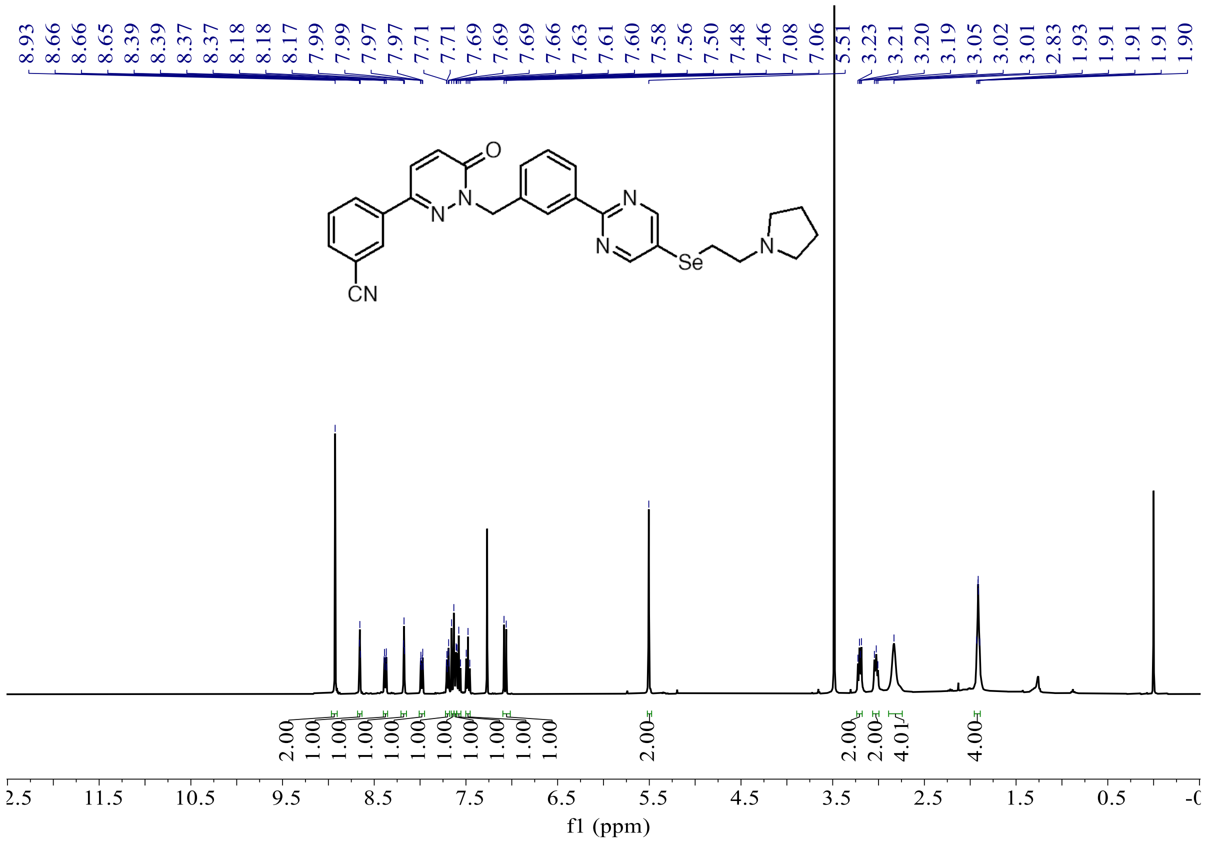

Figure S13. ^{1}H NMR of compound **8g** ($CDCl_3$, 400 MHz)

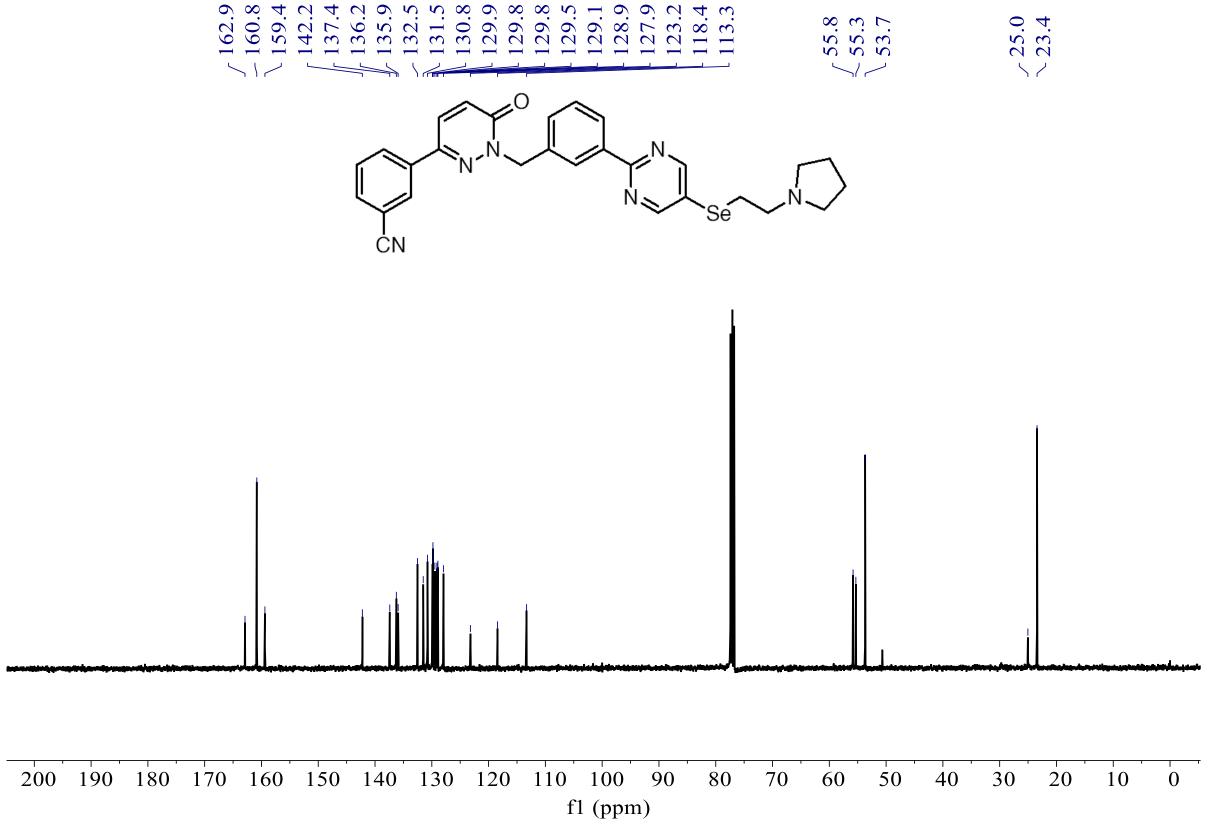

Figure S14. ^{13}C NMR of compound **8g** ($CDCl_3$, 100 MHz)

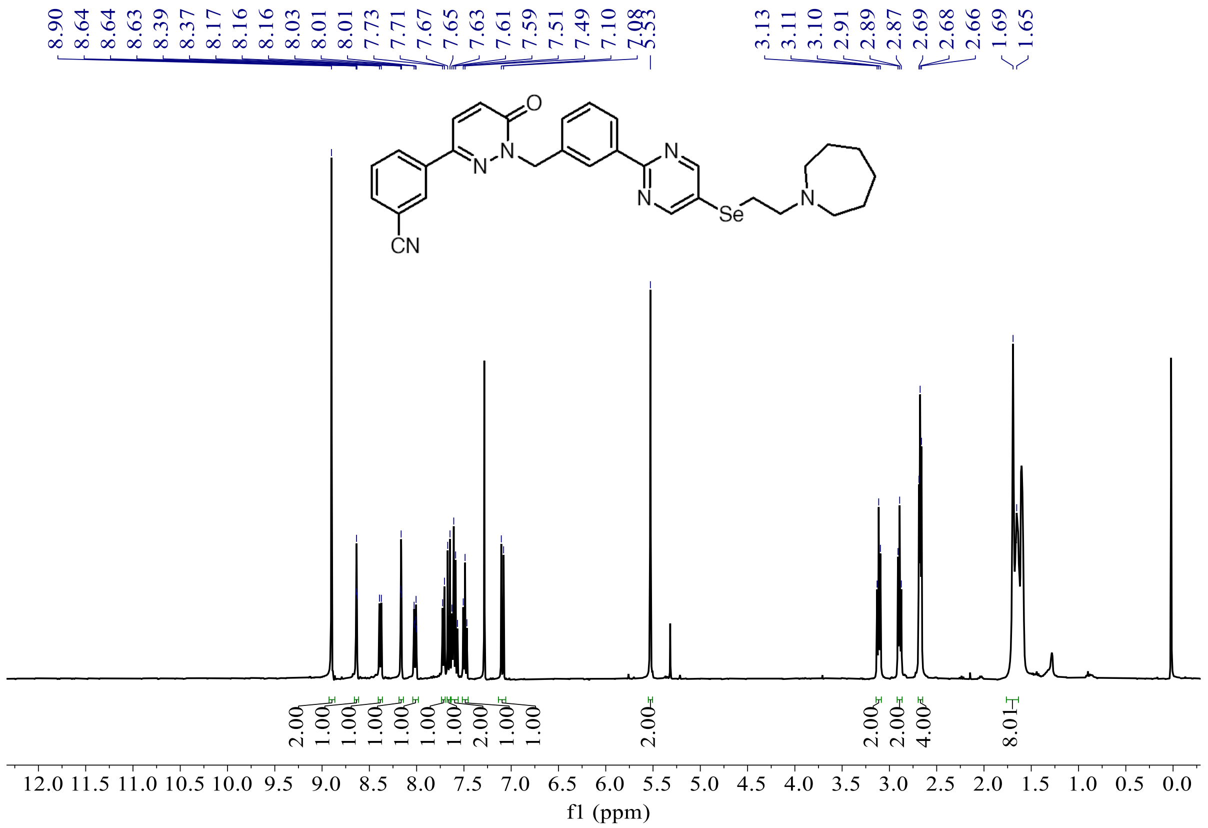

Figure S15. ^{1}H NMR of compound **8h** ($CDCl_3$, 400 MHz)

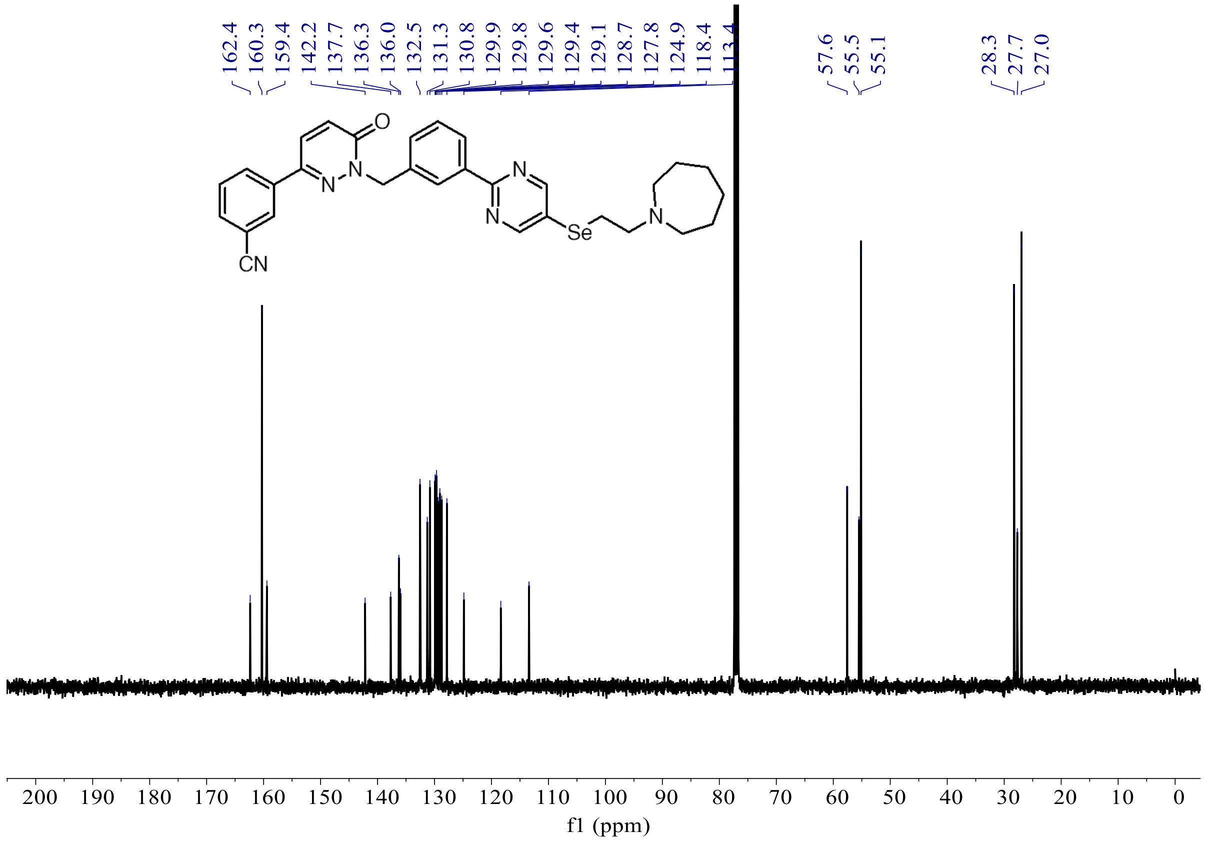

Figure S16. ^{13}C NMR of compound **8h** ($CDCl_3$, 100 MHz)

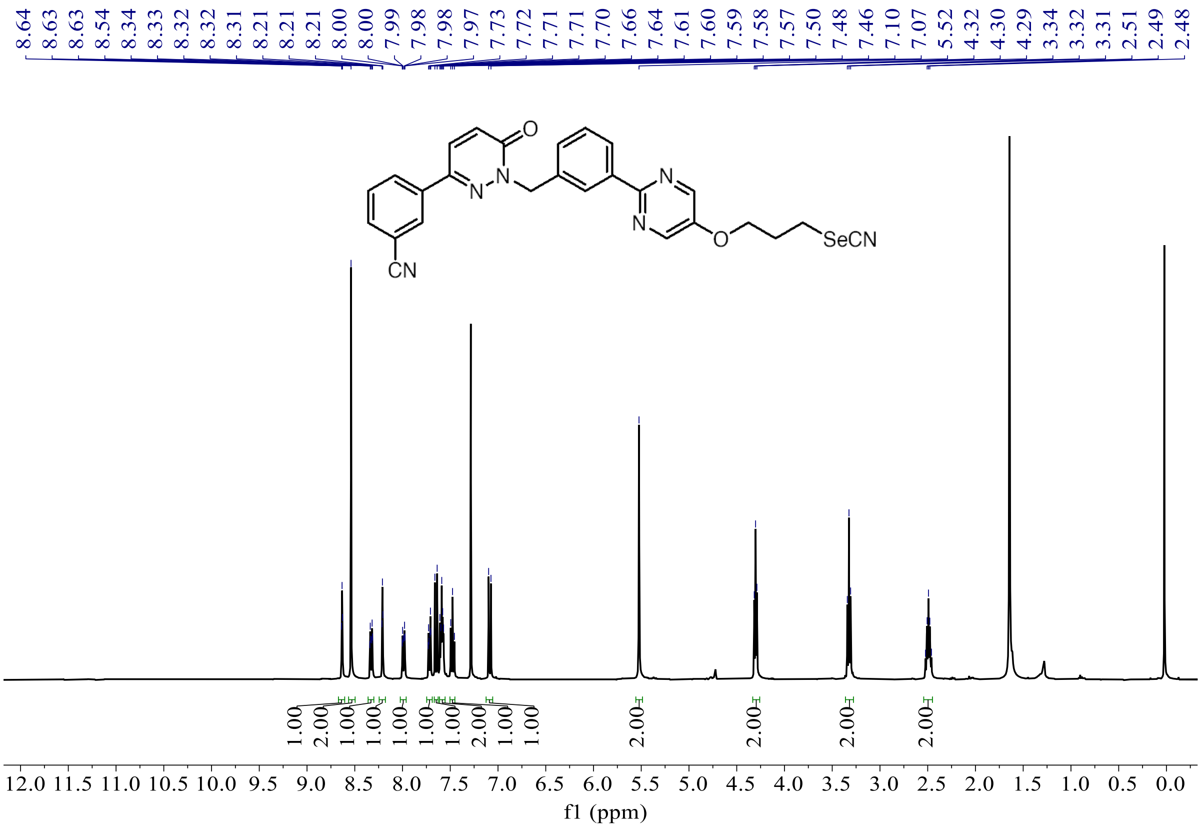

Figure S17. ^{1}H NMR of compound **9a** ($CDCl_3$, 400 MHz)

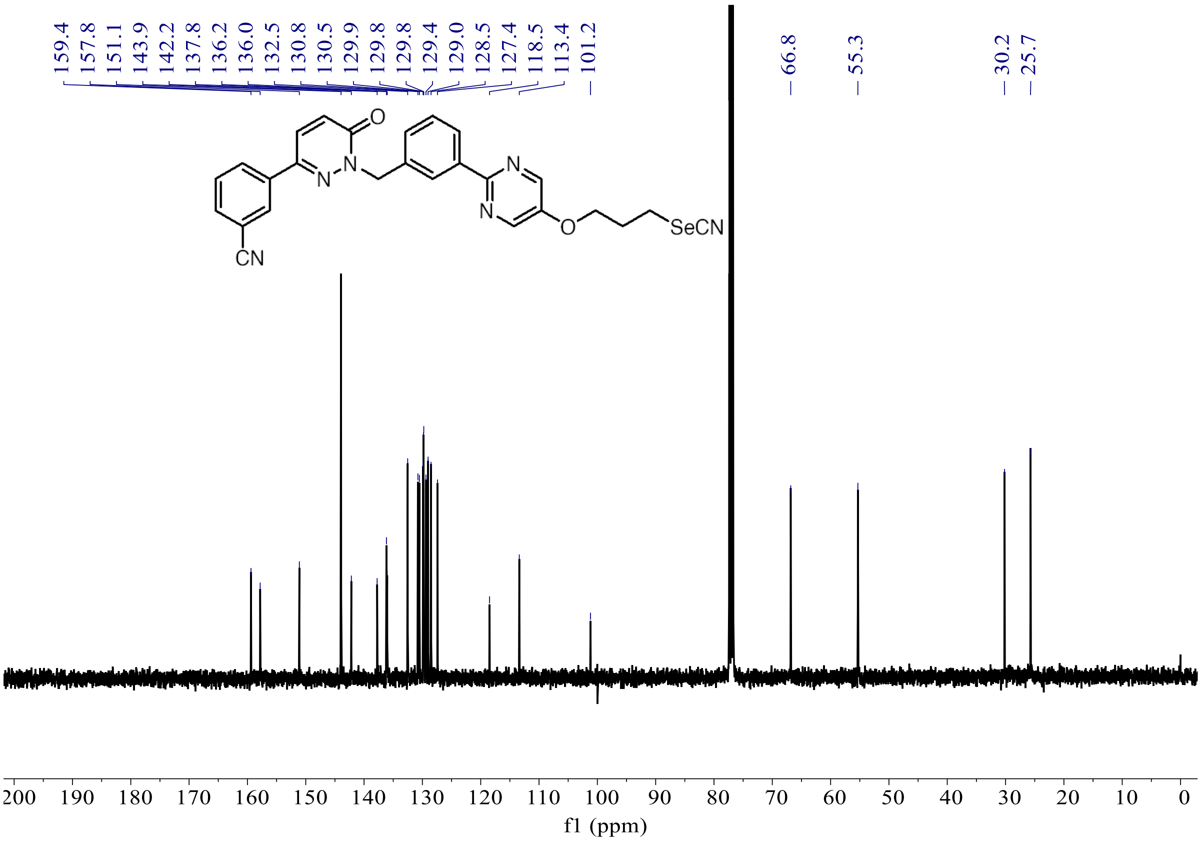

Figure S18. ^{13}C NMR of compound **9a** ($CDCl_3$, 100 MHz)

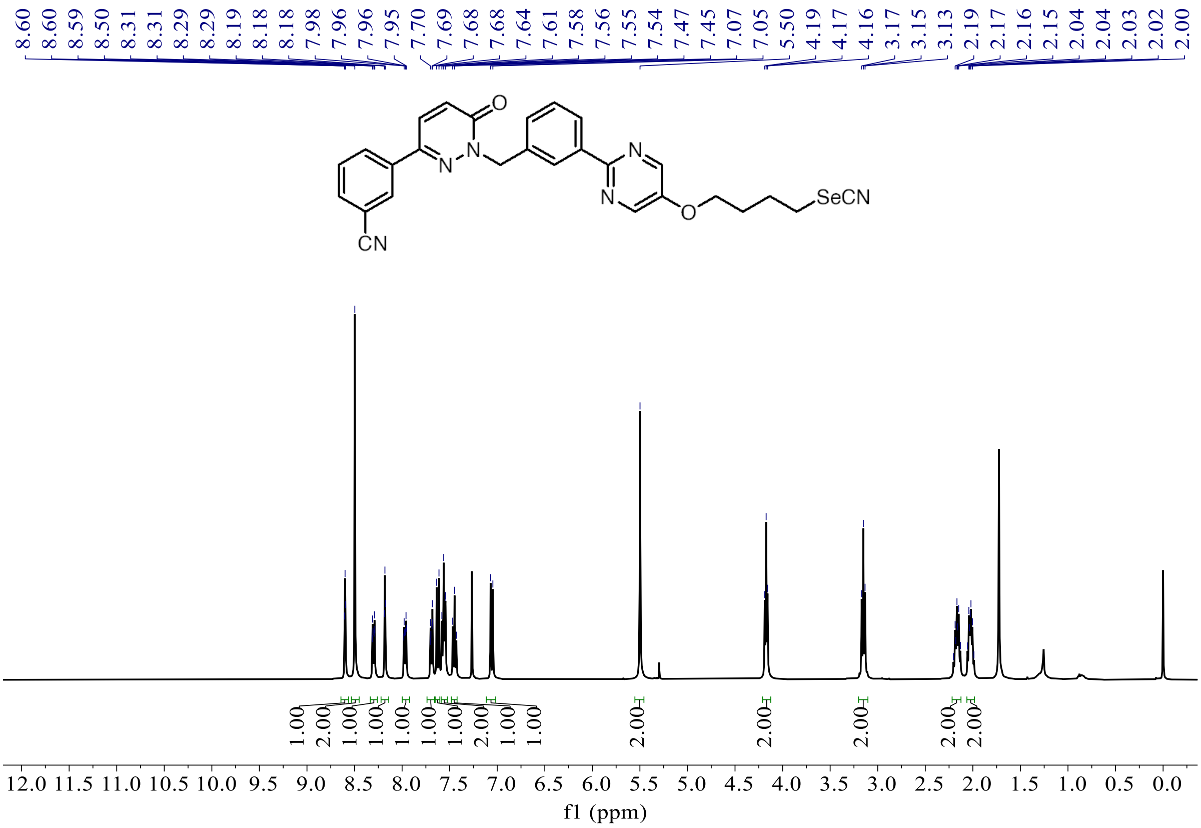

Figure S19. ^{1}H NMR of compound **9b** ($CDCl_3$, 400 MHz)

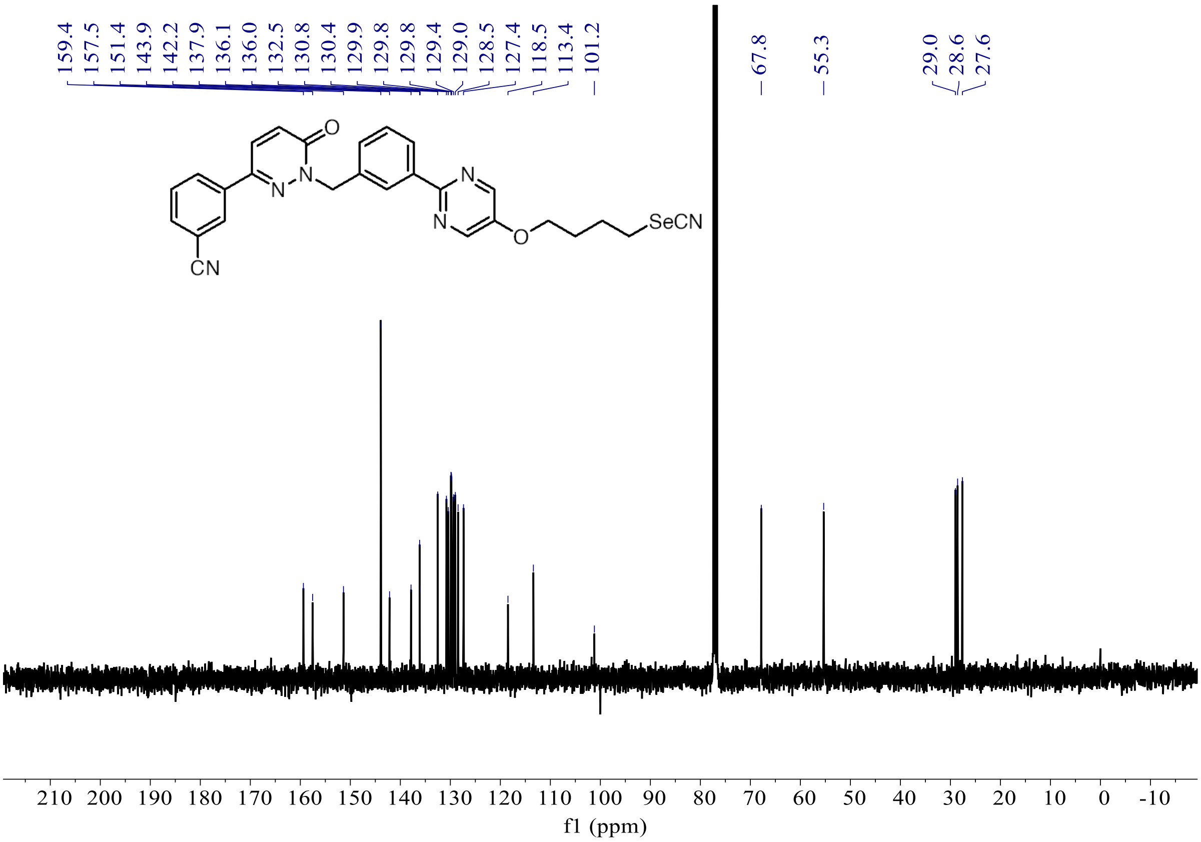

Figure S20. ^{13}C NMR of compound **9b** ($CDCl_3$, 100 MHz)

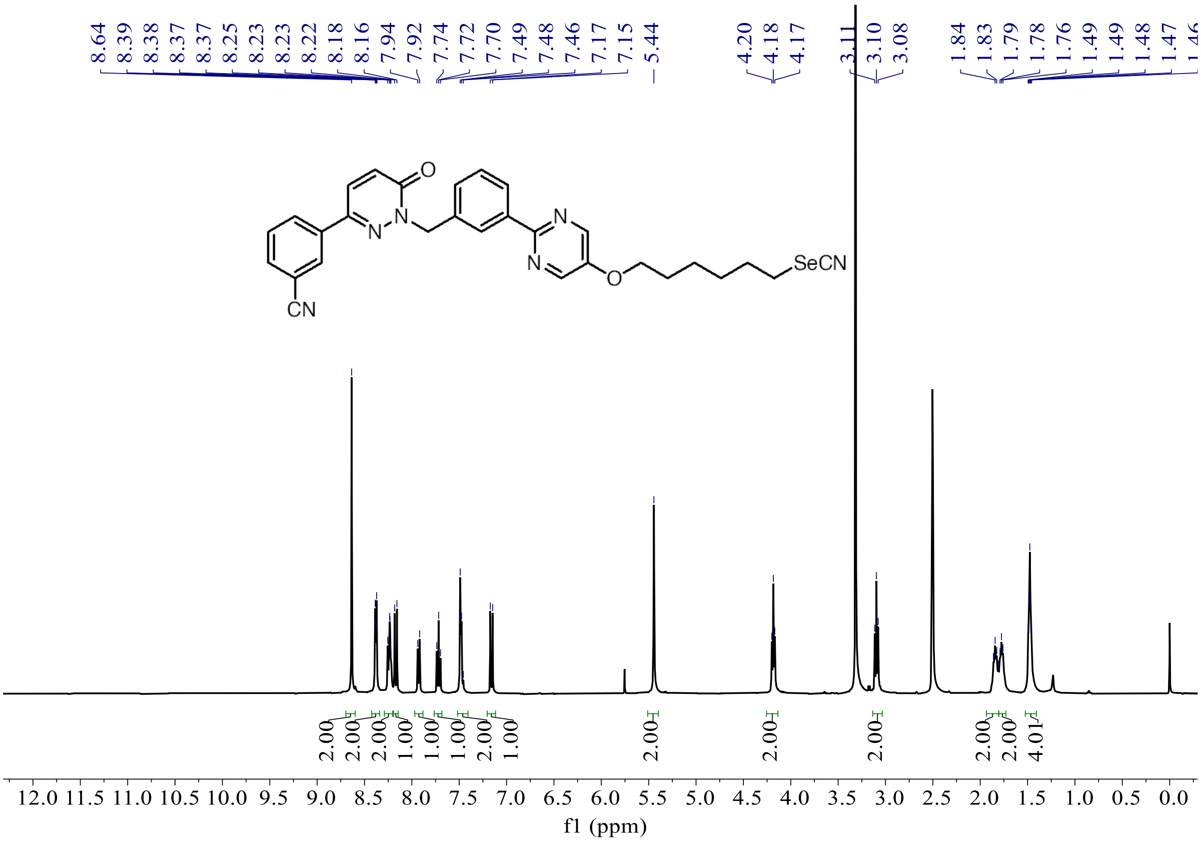

Figure S21. ^{1}H NMR of compound **9c** (DMSO-*d*$_6$, 400 MHz)

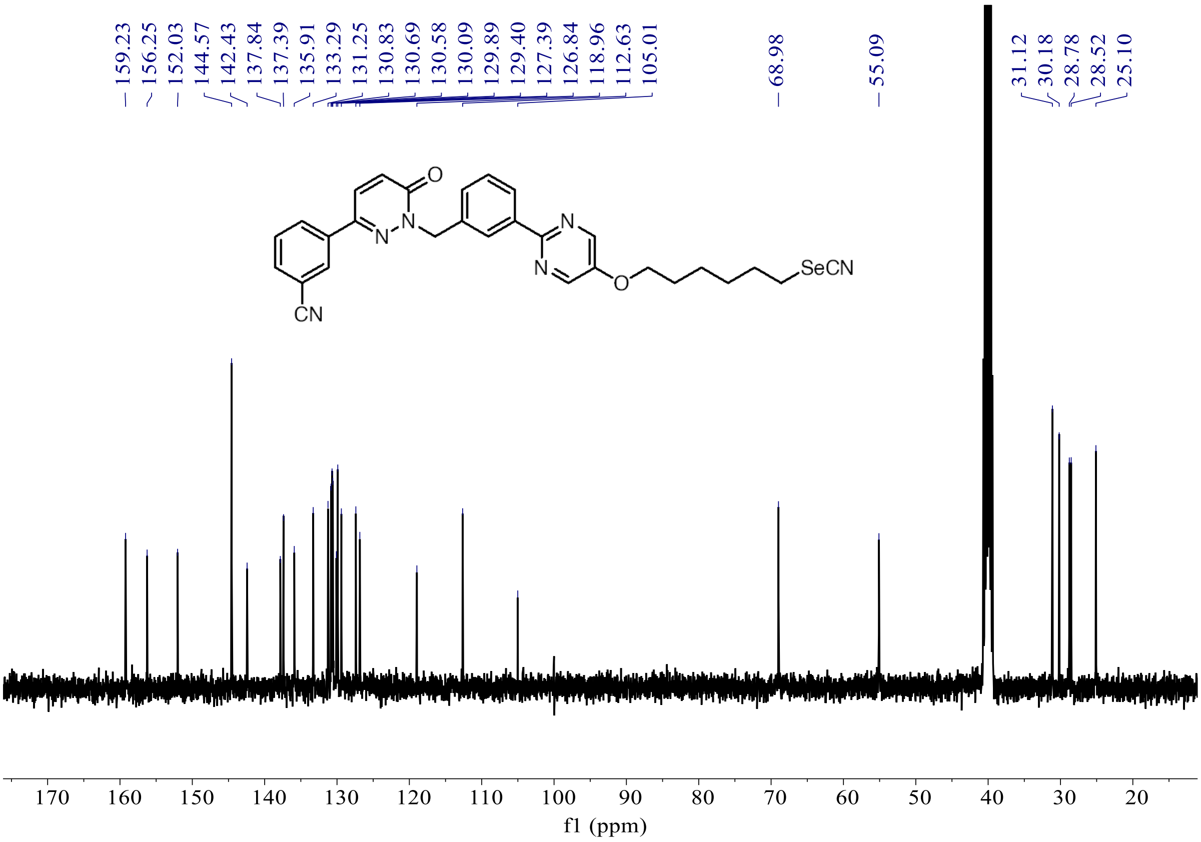

Figure S22. ^{13}C NMR of compound **9c** (DMSO-*d*$_6$, 100 MHz)

3 HPLC Chromatograms of Compounds 8a-h and 9a-c

Compound **8a**

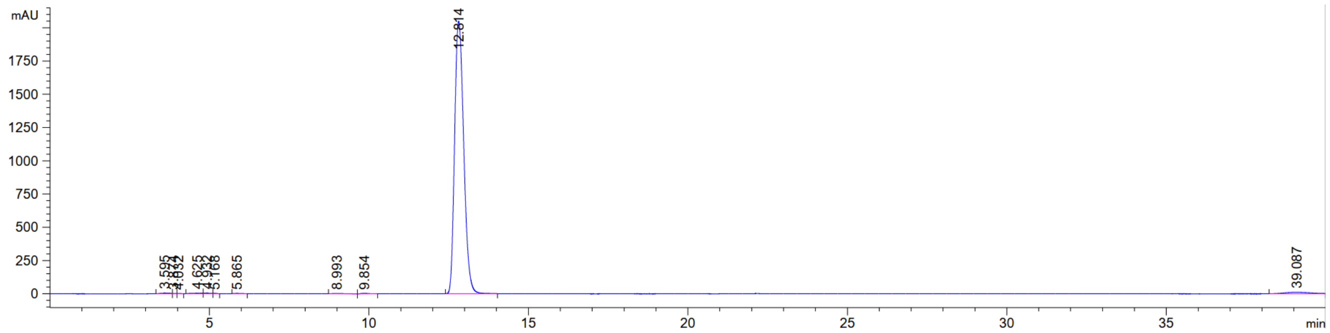

No. #	Retention time (tR) [min]	Type	Peak width [min]	Peak area [mAU*s]	Peak height [mAU]	Peak area% %
1	3.595	BV	0.2108	106.28583	6.20616	0.2542
2	3.874	VB	0.0900	14.94803	2.23598	0.0358
3	4.032	BB	0.1105	15.76241	1.88613	0.0377
4	4.625	BV	0.2652	118.43855	5.41509	0.2833
5	4.932	VV	0.1770	80.69726	5.74215	0.1930
6	5.168	VB	0.1039	23.43851	3.33726	0.0561
7	5.865	BB	0.1525	26.39571	2.06010	0.0631
8	8.993	BV	0.3138	76.57977	2.88436	0.1832
9	9.854	VB	0.1950	65.88264	4.31803	0.1576
10	12.814	BB	0.3151	4.06012e4	2049.96240	97.1163
11	39.087	BBA	0.6975	677.17212	11.42559	1.6198

Compound **8b**

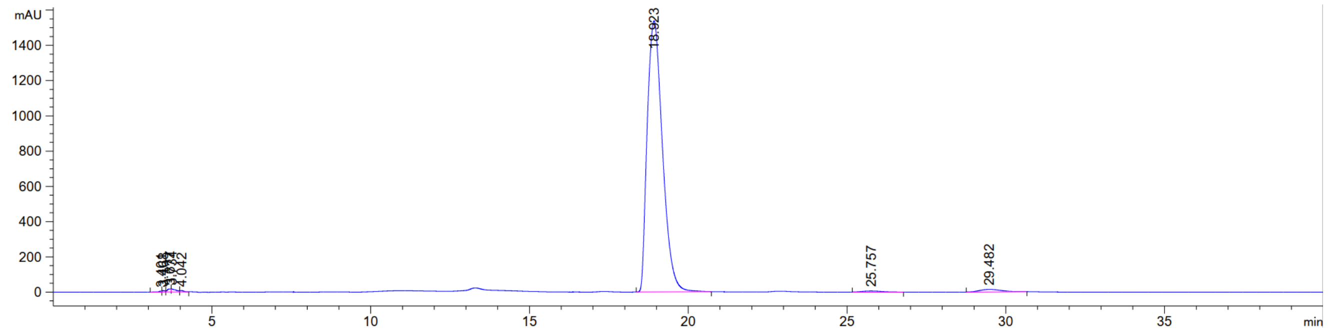

No. #	Retention time (tR) [min]	Type	Peak width [min]	Peak area [mAU*s]	Peak height [mAU]	Peak area% %
1	3.401	BV	0.0914	35.65413	5.74156	0.0676
2	3.468	VV	0.0841	52.40971	8.36131	0.0994
3	3.677	VV	0.1089	140.23622	17.42970	0.2659
4	3.734	VV	0.1325	179.84424	16.99470	0.3410
5	4.042	VB	0.1176	71.28277	8.43849	0.1352
6	18.923	BB	0.5233	5.13232e4	1538.21045	97.3271
7	25.757	BB	0.4864	253.65410	6.10967	0.4810
8	29.482	BB	0.5724	676.38702	14.03046	1.2827

Compound **8c**

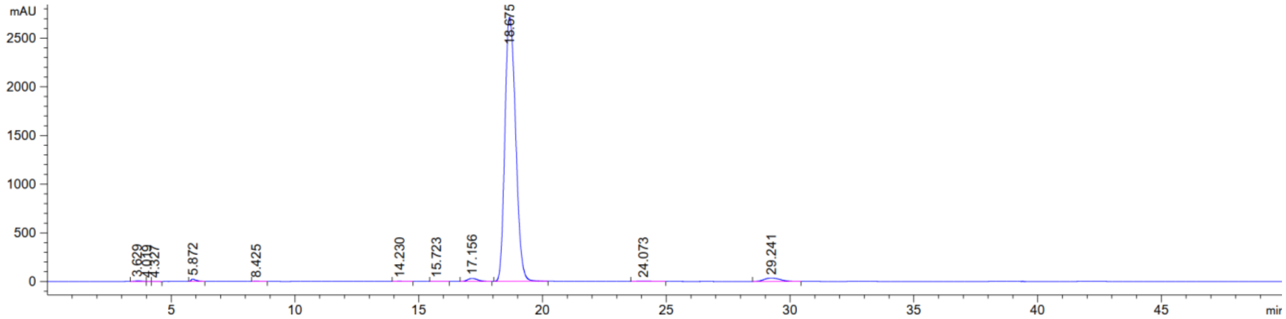

No. #	Retention time (t_R) [min]	Type	Peak width [min]	Peak area [mAU*s]	Peak height [mAU]	Peak area% %
1	3.629	BV	0.2633	154.93164	7.19169	0.1819
2	4.019	VB	0.1111	20.51091	2.34263	0.0241
3	4.327	BV	0.2103	37.65500	2.10949	0.0442
4	5.872	BB	0.2003	330.05197	24.02873	0.3876
5	8.425	VB	0.2037	30.55770	2.36533	0.0359
6	14.230	BB	0.2897	47.37447	1.93443	0.0556
7	15.723	BB	0.2701	34.41406	1.50878	0.0404
8	17.156	BB	0.4522	875.24738	30.28609	1.0277
9	18.675	BB	0.4213	8.19185e4	2713.04541	96.1895
10	24.073	BB	0.4888	128.60037	3.18011	0.1510
11	29.241	BB	0.6783	1585.77356	34.82467	1.8620

Compound **8d**

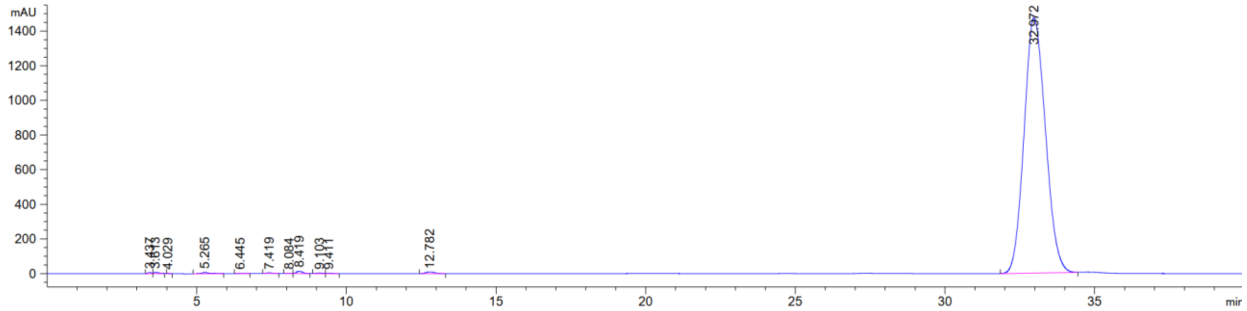

No. #	Retention time (t_R) [min]	Type	Peak width [min]	Peak area [mAU*s]	Peak height [mAU]	Peak area% %
1	3.437	BV	0.1221	49.34755	5.22921	0.0668
2	3.613	VV	0.1683	83.84934	6.33807	0.1135
3	4.029	VB	0.0804	8.95464	1.50499	0.0121
4	5.265	VB	0.2486	153.08983	7.84752	0.2073
5	6.445	BB	0.1352	12.35920	1.11642	0.0167
6	7.419	BB	0.1652	47.29662	3.77517	0.0640
7	8.084	BV	0.1407	13.28651	1.11759	0.0180
8	8.419	VB	0.2119	181.09404	13.20951	0.2452
9	9.103	BV	0.1992	47.14421	2.89198	0.0638
10	9.411	VB	0.1446	24.47696	2.07492	0.0331
11	12.782	BB	0.2601	182.94115	9.11823	0.2477
12	32.972	BB	0.7784	7.30415e4	1473.31604	98.9115

Compound **8e**

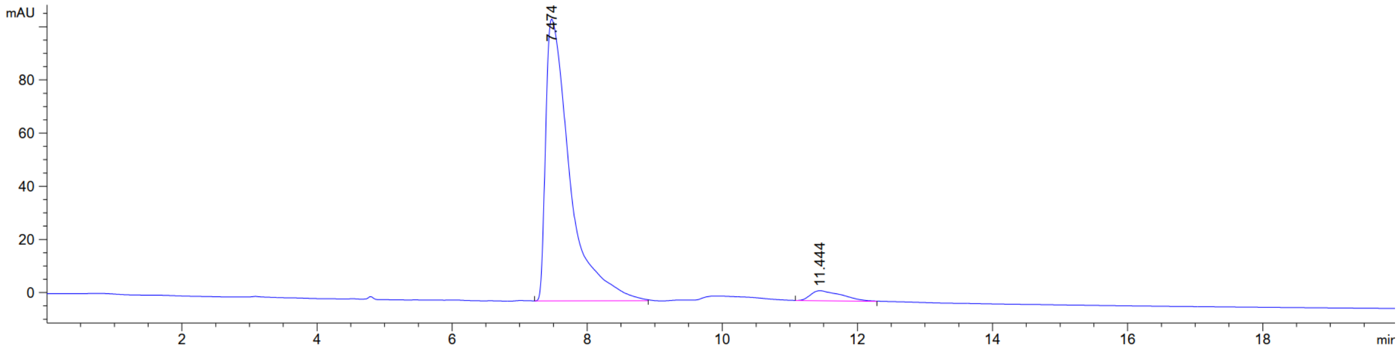

No. #	Retention time (t_R) [min]	Type	Peak width [min]	Peak area [mAU*s]	Peak height [mAU]	Peak area% %
1	7.474	BB	0.3824	2638.03296	105.97525	95.6582
2	11.444	BB	0.4437	119.73682	3.73768	4.3418

Compound **8f**

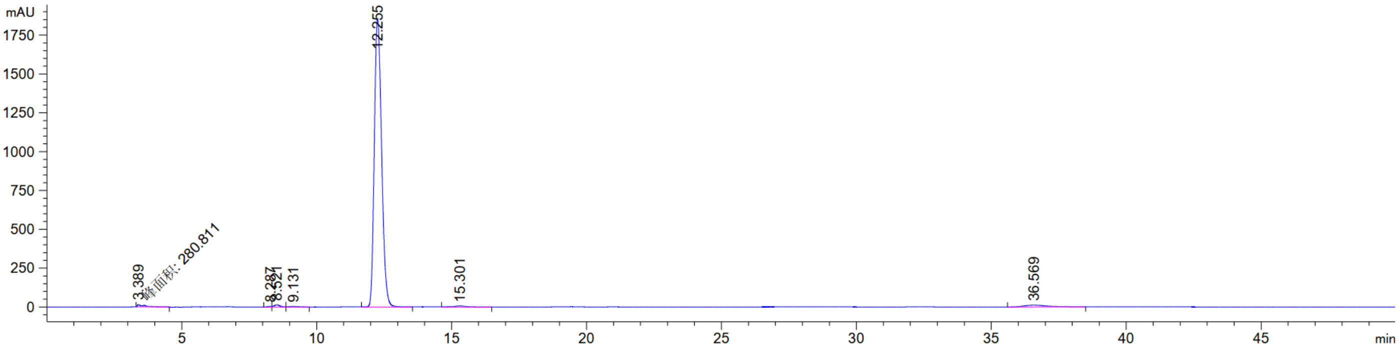

No. #	Retention time (t_R) [min]	Type	Peak width [min]	Peak area [mAU*s]	Peak height [mAU]	Peak area% %
1	3.389	MM	0.2995	280.81137	15.62765	0.7693
2	8.287	BV	0.1385	38.23758	4.26805	0.1047
3	8.521	VV	0.2288	210.19678	14.02681	0.5758
4	9.131	VV	0.3711	105.48045	4.00720	0.2890
5	12.255	VB	0.2957	3.48907e4	1853.01306	95.5803
6	15.301	VB	0.4729	226.84782	6.94824	0.6214
7	36.569	BB	0.8952	751.79474	11.93062	2.0595

Compound **8g**

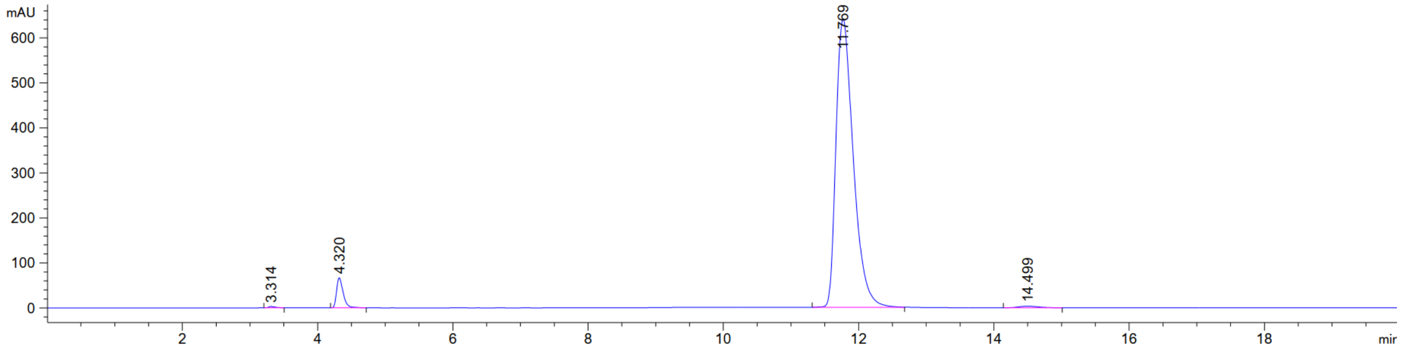

No. #	Retention time (t_R) [min]	Type	Peak width [min]	Peak area [mAU*s]	Peak height [mAU]	Peak area% %
1	3.314	BB	0.1069	21.83522	3.00114	0.1824
2	4.320	VB	0.1038	457.67694	66.94666	3.8233
3	11.769	BB	0.2757	1.14162e4	641.13904	95.3682
4	14.499	BB	0.3169	74.94468	3.62893	0.6261

Compound **8h**

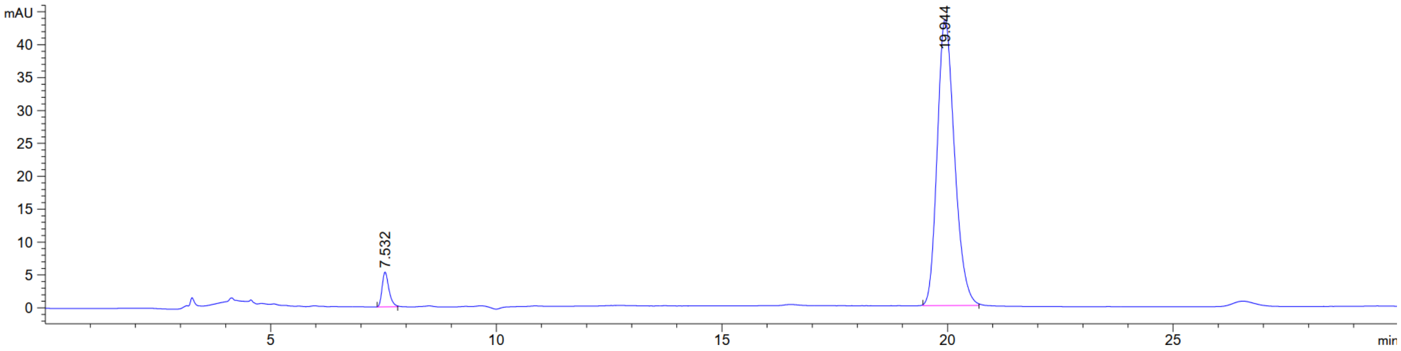

No. #	Retention time (t_R) [min]	Type	Peak width [min]	Peak area [mAU*s]	Peak height [mAU]	Peak area% %
1	7.532	BB	0.1583	55.43755	5.28935	4.5714
2	19.944	BB	0.4105	1157.26160	43.48883	95.4286

Compound **9a**

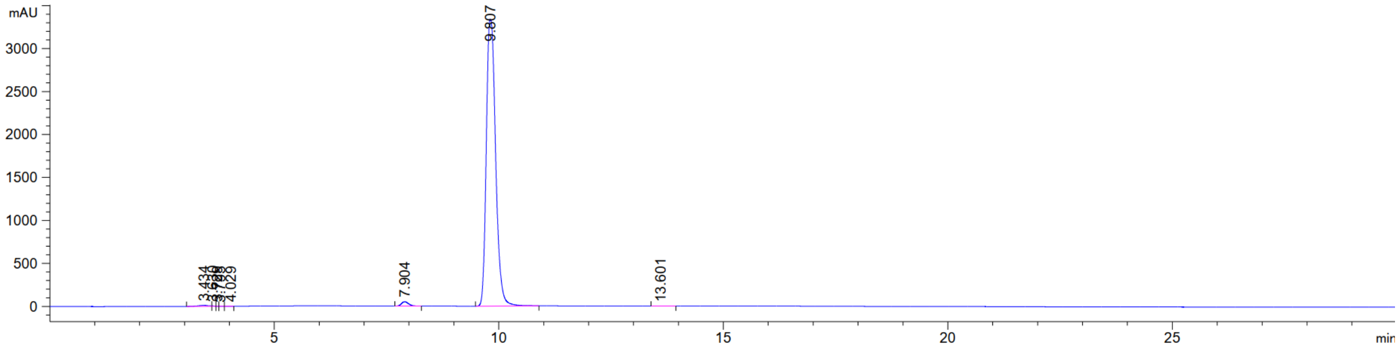

No. #	Retention time (t_R) [min]	Type	Peak width [min]	Peak area [mAU*s]	Peak height [mAU]	Peak area% %
1	3.434	BV	0.1976	184.19438	12.10298	0.3773
2	3.630	VV	0.0454	17.71718	4.99214	0.0363
3	3.729	VV	0.0441	5.45459	1.67108	0.0112
4	3.795	VV	0.0690	7.30620	1.39739	0.0150
5	4.029	VV	0.1188	10.25241	1.09913	0.0210
6	7.904	BB	0.1711	553.64301	50.42330	1.1340
7	9.807	BB	0.1739	4.80295e4	3337.69482	98.3728
8	13.601	BB	0.1747	15.87782	1.09143	0.0325

Compound **9b**

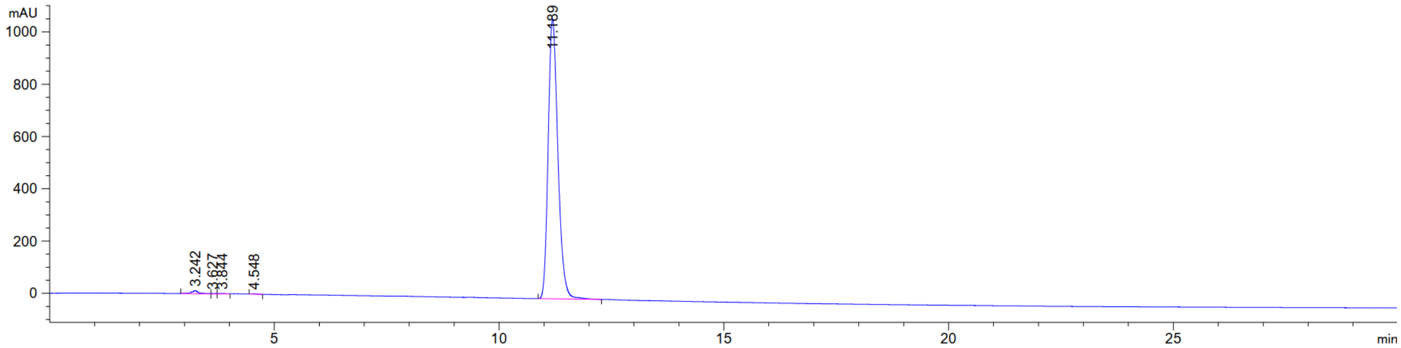

No. #	Retention time (t_R) [min]	Type	Peak width [min]	Peak area [mAU*s]	Peak height [mAU]	Peak area% %
1	3.242	BV	0.1551	130.91510	11.47630	0.8047
2	3.627	VB	0.0918	7.96512	1.16417	0.0490
3	3.844	BV	0.1691	19.37383	1.49551	0.1191
4	4.548	BB	0.0957	9.62702	1.50285	0.0592
5	11.189	BB	0.2317	1.61018e4	1068.95801	98.9681

Compound **9c**

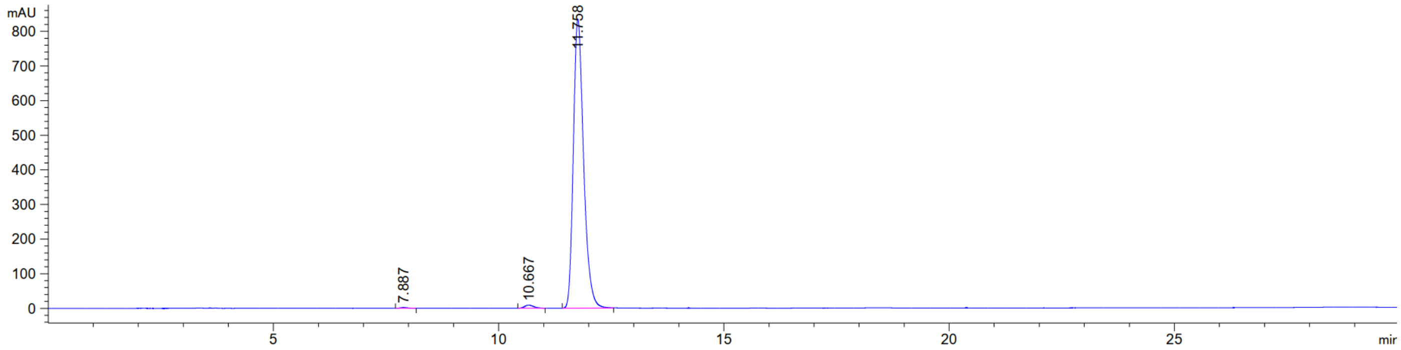

No. #	Retention time (t_R) [min]	Type	Peak width [min]	Peak area [mAU*s]	Peak height [mAU]	Peak area% %
1	7.887	BB	0.1408	26.55102	2.54236	0.1991
2	10.667	BB	0.2068	124.97034	8.95521	0.9373
3	11.758	BB	0.2423	1.31822e4	834.39655	98.8636

4 Uncropped and Unadjusted Images of Western Blot

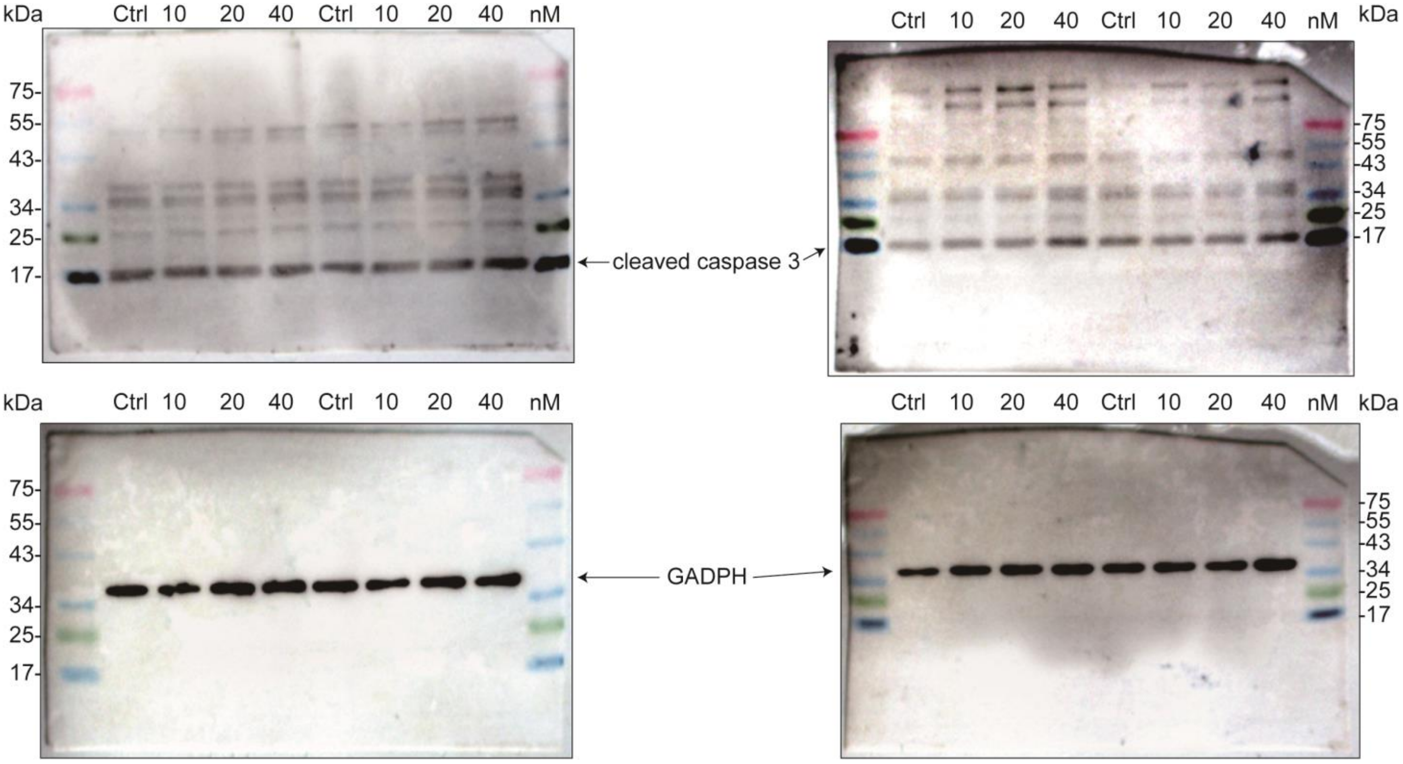

Figure S23. Expanded and uncropped western blot panels of cleaved caspase 3 in MHCC97H cells after treatment with compound **8b** (10, 20 and 40 nM) for 24 h.
